# Changing substrate specificity and iteration of amino acid chain elongation in glucosinolate biosynthesis through targeted mutagenesis of *Arabidopsis* methylthioalkylmalate synthase 1

**DOI:** 10.1042/BSR20190446

**Published:** 2019-07-02

**Authors:** Annette Petersen, Lea Gram Hansen, Nadia Mirza, Christoph Crocoll, Osman Mirza, Barbara Ann Halkier

**Affiliations:** 1 DynaMo Center, Department of Plant and Environmental Sciences, Faculty of Science, University of Copenhagen, Frederiksberg, Denmark; 2 Copenhagen Plant Science Centre, Department of Plant and Environmental Sciences, Faculty of Science, University of Copenhagen, Frederiksberg, Denmark; 3 The Novo Nordisk Foundation Center for Biosustainability, Technical University of Denmark, Lyngby, Denmark; 4 NanoTemper Technologies ApS, Taastrup, Denmark; 5 Department of Drug Design and Pharmacology, Faculty of Health and Medical Sciences, University of Copenhagen, Copenhagen, Denmark

**Keywords:** biotechnology, enzyme activity, Escherichia coli, structural biology

## Abstract

Methylthioalkylmalate synthases catalyse the committing step of amino acid chain elongation in glucosinolate biosynthesis. As such, this group of enzymes plays an important role in determining the glucosinolate composition of Brassicaceae species, including *Arabidopsis thaliana*. Based on protein structure modelling of MAM1 from *A. thaliana* and analysis of 57 MAM sequences from Brassicaceae species, we identified four polymorphic residues likely to interact with the 2-oxo acid substrate. Through site-directed mutagenesis, the natural variation in these residues and the effect on product composition were investigated. Fifteen MAM1 variants as well as the native MAM1 and MAM3 from *A. thaliana* were characterised by heterologous expression of the glucosinolate chain elongation pathway in *Escherichia coli*. Detected products derived from leucine, methionine or phenylalanine were elongated with up to six methylene groups. Product profile and accumulation were changed in 14 of the variants, demonstrating the relevance of the identified residues. The majority of the single amino acid substitutions decreased the length of methionine-derived products, while approximately half of the substitutions increased the phenylalanine-derived products. Combining two substitutions enabled the MAM1 variant to increase the number of elongation rounds of methionine from three to four. Notably, characterisation of the native MAMs indicated that MAM1 and not MAM3 is responsible for homophenylalanine production. This hypothesis was confirmed by glucosinolate analysis in *mam1* and *mam3* mutants of *A. thaliana*.

## Introduction

**Figure 1:**
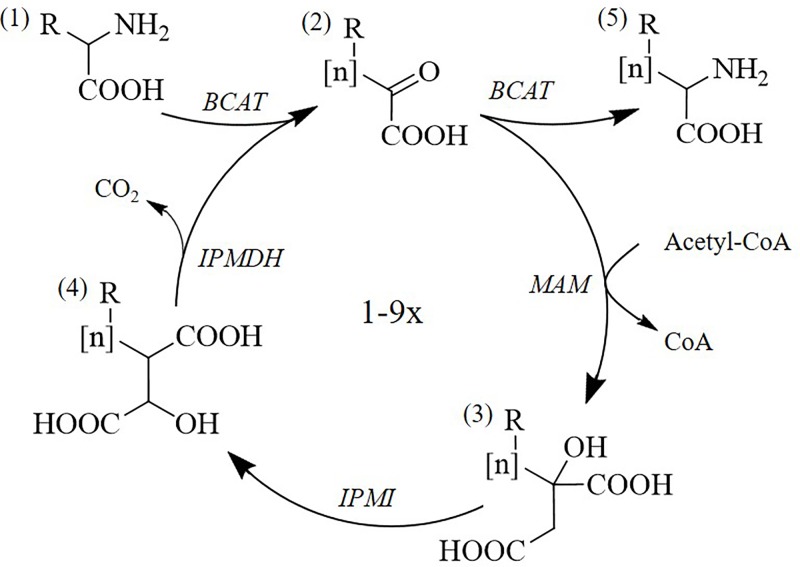
Schematic view of the chain elongation pathway in glucosinolate biosynthesis The first step is deamination by a branched-chain aminotransferase (BCAT), followed by a three-step cycle: condensation by a MAM, isomerisation by an isopropylmalate isomerase (IPMI) and oxidative decarboxylation by an isopropylmalate dehydrogenase (IPMDH). At this point the intermediate will either go through another cycle or exit by transamination by a BCAT. The pathway intermediates are (1) amino acid, (2) 2-oxo acid, (3) 2-alkylmalic acid, (4) 3-alkylmalic acid and (5) chain-elongated amino acid.

Glucosinolates are amino acid-derived chemical defence compounds characteristic of the Brassicales order [1]. In biosynthesis of methionine-derived aliphatic glucosinolates, the chain elongation process generates side chain-elongated methionine derivatives as precursor for the corresponding glucosinolates [2]. In the iterative process, one methylene group is added in each cycle (Figure 1). This process evolved from the non-iterative leucine biosynthesis in which one methylene group is added to the side chain of valine [3–6]. The methionine elongation process has uniquely evolved the ability to add up to nine methylene groups [7]. Interestingly, the glucosinolate structural diversity provides differences in biological function with impact on disease resistance and plant fitness [8].

All enzymes of methionine chain elongation are known in *Arabidopsis thaliana* and the methylthioalkylmalate synthases (MAMs) determine the number of elongation cycles [5,9–11]. Previous studies investigated the evolutionary process from the isopropylmalate synthase (IPMS) to MAM through domain-scale modifications and site-directed mutagenesis. They successfully changed substrate preference of IPMS2 to resemble that of MAM1, thereby identifying a set of residues important for substrate specificity [12].

IPMSs and MAMs both condensate 2-oxo acids with acetyl-CoA, which places them in the Claisen-like condensation (CC-like) subgroup of the DRE-TIM metallolyase superfamily [13]. Two IPMS enzymes have been crystallised from this subgroup: *Mycobacterium tuberculosis* (MtIPMS, [14] and *Neisseria meningitidis* (NmIPMS, [15]. The crystal structures indicate that the IPMS enzymes form homo-dimers. Each monomer has two domains with a catalytic site and the LeuA regulatory domain that inhibits activity in the presence of leucine [16–18]. This regulatory domain is lost in MAM enzymes [12]. Previous studies have shown that the LeuA domain is critical for the quaternary structure of IPMS enzymes. Removal of this domain from IPMS2 resulted in a complete loss of quaternary structure and adding the domain to MAM1 and MAM3 caused the otherwise monomeric enzymes to form both dimers and tetramers [12]. The authors hypothesised that losing the quaternary structure increased the active site of MAM synthases and thereby allowed for bulkier substrates. Individual residues were identified as important for evolution of the MAM synthases. Eleven residues that have been under positive selection during the iterative adaptation of the MAM synthases are Glu96, Ser98, Ile138, Cys165, Thr173, Leu177, Val187, Thr257, Ile258, Gly259 and Val289 according to the numbering in MAM1 of *A. thaliana* [5]. Based on MAM homology models, five residues are proposed to bind the substrate: Arg93, Asp94, Thr261, His292 and His292. Additional six residues are predicted to hydrophobically interact with the substrate: Ile162, Leu186, Gly229, Glu231, Thr257 and Gly259 [12].

Sequencing of the MAM gene cluster of several *Arabidopsis* species reveal three distinct clades of MAMs: MAMa, MAMb and MAMc [5]. In *A. thaliana*, MAM1 and MAM2 are homologues of MAMa, while MAM3 is a homologue of MAMb [10]. MAMc was lost in *A. thaliana* but is still found in e.g. *A. lyrata.* MAM3 has a broader substrate specificity than MAM1, which in turn is broader than MAM2 [5,10,11]. MAM2 catalyses only the first round of elongation and is found in accessions rich in glucosinolates derived from homomethionine (e.g. L*er*-0) [5], while MAM1 catalyses C3–C5 and is found in accessions accumulating glucosinolates derived from dihomomethionine (e.g. Col-0) [9]. MAM3 catalyses all lengths from C3–C8 and thus is present in – but not restricted to – all accessions with glucosinolates derived from tetra-, penta- and hexahomomethionine [10].

Quantitative trait loci mapping has implicated the GS-ELONG locus in not only controlling chain elongation of methionine but also phenylalanine, from which 2-phenylethyl glucosinolate is derived [19]. When characterising MAM3 in *in vitro* assays, chain elongation of methionine as well as leucine, isoleucine and phenylalanine were detected [10]. Based on these findings, MAM3 was proposed to be responsible for elongation of phenylalanine *in planta*. When the chain elongation pathway of *A. thaliana*, i.e. *MAM1, BCAT4, IPMI-LSU1, IPMI-SSU3* and *IPMDH1*, was expressed in *Escherichia coli*, methionine- and leucine-derived products elongated by either one or two methylene groups, were produced [20]. Similar results were obtained when the chain elongation pathway was expressed in *Nicotiana benthamiana* [21,22]. As leucine-derived glucosinolates are not found in the native *A. thaliana* plant, the results indicate that MAM synthases lose specificity when expressed in heterologous hosts.

The Brassicaceae is a large family with over 3700 species divided into 338 genera and 19 tribes [23]. Based on a phylogenetic analysis of 114 sequences within the family, a division into three lineages was proposed [24]. Several whole genome and local tandem duplications combined with subsequent deletions have resulted in a poor resolution of the MAM family in Brassicaceae. Furthermore, predicting activity from sequence data alone can be difficult and imprecise [25], as evident from IPMS enzymes, which show nearly identical substrate specificity despite having a relatively moderate sequence identity [18]. Understanding how the substrate specificity of MAMs controls the iterative chain elongation process is important from a basic science as well as a bioengineering perspective.

The present study investigates the impact of polymorphic residues specific to the MAM enzymes on substrate specificity and number of iterations. A well-established method for this purpose is site-directed mutagenesis on evolutionarily conserved residues [17]. We used this approach to create a library of MAM1 variants designed to have changed substrate specificity. Based on a multiple alignment of MAM synthase sequences and IPMS crystal structures, evolutionary important residues were predicted and targeted for mutation. Since the chain elongation pathway has been successfully expressed in *E. coli* previously [20], we chose to characterise the MAM1 variants *in vivo* using this host. Four residues were mutated individually or in combinations, and a total of 15 variants were characterised. Substrate specificity and number of elongations were compared with the native MAM1, MAM3 and IPMS2 from *A. thaliana*. Targeted proteomics was used to monitor expression of the proteins in *E. coli* and to eliminate changes in product or titres caused by unequal expression of proteins between the different *E. coli* strains.

## Materials and methods

### Bioinformatics and structural modelling

Protein sequences of MAM1, MAM2, MAM3, IPMS1 and IPMS2 from *A. thaliana* were collected from TAIR (Arabidopsis.org). Additional MAM sequences were identified through BLASTp and tBLASTn searches on NCBI using the MAM sequences. MAMs from *Camelina sativa, Brassica rapa, Brassica oleracea, Brassica napus, Eutrema salsugineum, Capsella rubella, Arabidopsis lyrata, Arabidopsis cebennensis, Boechera divaricarpa, Raphanus sativus* and *Eruca vesicaria* were collected and used in an alignment (Supplementary Figures S1 and S2). Sequences with large deletions were removed. Alignments were made using the slow algorithm in CLC Main Workbench 8.0.1 (www.qiagenbioinformatics.com). A homology model of the MAM1 protein structure was constructed based on the *N. meningitis* IPMS crystal structure (PDB-ID 3RMJ) [15] using BLAST [26] to align the two sequences (40.7% identity) and Modeller to create the model [27]. Structural analysis were done in PyMOL (the PyMOL Molecular Graphics System, version 1.5.0.4 Schrödinger, LLC).

### Generation of expression constructs for MAM1 variants

**Figure 2:**
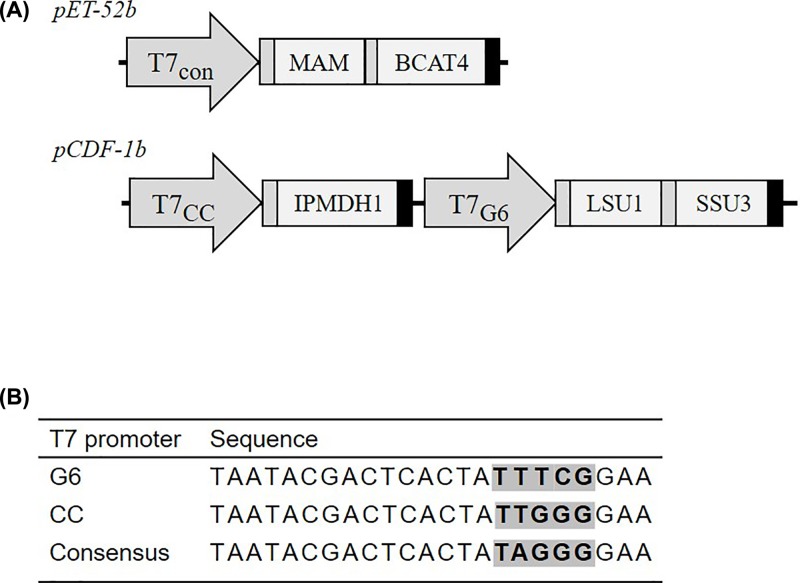
Design of expression constructs containing the chain elongation pathway of *A. thaliana* (**A**) The two constructs used in combination to express the biosynthetic genes. The MAM gene is replaced with native or mutated variants of MAM1 and MAM3 as well as IPMS2. Different T7 promoters were used as indicated in the arrows. Ribosomal binding sites, genes and terminators are indicated as dark grey, light grey and black boxes, respectively. (**B**) Promoters used in this work. Sequence differences are highlighted.

All constructs were cloned by USER cloning [28]. As described in [21], the chain elongation pathway genes were expressed from two plasmids; *BCAT4* and *MAM1* on pET-52b (Novagen®, Merck, #71554) and *IPMI-LSU1, IPMI-SSU3* and *IPMDH1* on pCDF-1b (Novagen®, Merck, #71330). Two changes were made to the T7 promoters of *IPMI* and *IPMDH1* compared with previous construct [20]. An alternative start codon was removed between the RBS and *IPMDH1* sequence, and the *IPMI* subunits were expressed from a separate promoter. Point mutations were introduced into *MAM1* by amplification with primers containing the desired mutation in the USER overhang (Supplementary Table S1). The CDS was amplified in two halves and fused by USER fusion [29]. The native *MAM1* sequence was replaced for the mutated MAM versions in the expression construct (Figure 2).

### Bacterial strains and cultivation

The *E. coli* NEB10B strain (NEB, #C3019H) was used for plasmid amplification and cloning purposes. The BL21 (DE3) strain (NEB, #C2527I) was used for expression. All transformations were done by heat-shock followed by one hour recovery in non-selective media before plating on LB agar plates (Luria–Bertani broth, Lennox, Duchefa, #L1703) containing 10 g tryptone, 5 g yeast extract, 5 g sodium chloride and 15g bactoagar per litre with 50 μg/ml spectinomycin and 100 μg/ml ampicillin. Expression experiments were performed in 24-well plates (ThermoFisher, #CS15124) containing 4 ml culture. Single colonies containing both chain elongation constructs were inoculated into LB media and grown overnight at 37°C, 220 rpm. Expression cultures were inoculated from the overnight culture into LB media with 50 μg/ml spectinomycin and 50 μg/ml carbenicillin. The cultures were started at a *D*_600_ (attenuance) at 0.1 and grown at 37°C, 220 rpm until *D*_600_ reached 0.6–0.8. Gene expression was then induced by addition of 1 mM IPTG (isopropyl d-β-1 thiogalactopyranoside) and the cultures were moved to 28°C, 220 rpm and harvested after 24 h.

### Metabolite extraction and LC–MS analysis

In *E. coli* cultures expressing the chain elongation pathway, only the medium was analysed for chain-elongated products, as the majority of the product were reported to be exported from the cells [20]. The samples were centrifuged at 13000 × *g* and supernatant diluted 25-fold in water, followed by 1:10 mixing with ^15^N^13^C-labelled amino acids (Algal amino acids 13C, 15N, Isotec, Miamisburg, U.S.A.) at a concentration of 10 μg/ml. Immediately before analysis, the samples were filtered through Durapore® 0.22 μm PVDF filter plates (Merck Millipore, Tullagreen, Ireland).

The LC–MS analysis was performed as previously described [20] with changes as detailed below. Chromatography was performed using an Advance UHPLC system (Bruker, Bremen, Germany) and a Zorbax Eclipse XDB-C18 column (100 × 3.0 mm, 1.8 μm, Agilent Technologies, Germany). The mobile phases A and B were formic acid (0.05% (v/v)) in water and acetonitrile (supplied with 0.05% (v/v) formic acid), respectively. The elution profile was: 0–1.2 min 3% B; 1.2–4.3 min 3–65% B; 4.3–4.4 min 65–100% B; 4.4–4.9 min 100% B, 4.9–5.0 min 100–3% B and 5.0–6.0 min 3% B. Mobile phase flow rate was 500 μl/min and column temperature was maintained at 40°C. An EVOQ Elite TripleQuad mass spectrometer with an electrospray ionisation source (ESI) (Bruker, Bremen, Germany) was coupled to the liquid chromatography. Pure standards were used to optimise the instrument parameters. The ion spray voltage was maintained at 3000 V in positive ionisation mode. Cone temperature was set to 350°C and cone gas flow to 20 psi. Heated probe temperature was set to 400°C and probe gas flow set to 50 psi. Nebulising gas was set to 60 psi and collision gas to 1.6 mTorr. Nitrogen was used as both cone gas and nebulising gas and argon as collision gas.

Multiple reaction monitoring (MRM) was used to monitor analyte parent ion to product ion transitions: MRM transitions for amino acids were chosen as previously described [30]. MRM transitions for chain-elongated amino acids were chosen as described in [20], and comparison to pure standards were used to verify the identification (Supplementary Figures S3 and S4). Both Q1 and Q3 quadrupoles were maintained at unit resolution. Bruker MS Workstation software (Version 8.2.1, Bruker, Bremen, Germany) was used for data acquisition and processing. A mixture of ^15^N^13^C-labelled amino acids was used as internal standard for quantification of protein amino acids. Details on transitions and collision energies can be found in Supplementary Table S2. Visualisation and statistical analyses were performed in R studio v1.0.153 [31] using [32–35].

### Protein extraction and targeted proteomics

#### Design of reference peptides

Proteotypic peptides were designed for each of the chain elongation enzymes to use as internal reference and a relative quantification of protein expression. Gene sequences were digested *in silico* using the SkyLine software [36]. The resulting list of peptides was then subjected to the following sorting criteria; (1) *m/z* < 1250, (2) cleavage site with only one arginine or lysine residue and (3) avoiding peptides with methionine or cysteine. All peptides fulfilling these requirements were ordered as synthetic isotopically labelled peptides (JPT, SpikeTides™). Peptides were resuspended by adding 100 μl 100 mM ammonium bicarbonate solution (pH 8.5) with 20% (v/v) acetonitrile and incubated gently shaking for 30 min at room temperature. Preferentially, each enzyme was covered by a minimum of two non-neighbouring peptides (Table 1).

**Table 1:** Peptides of chain elongation enzymes and the *E. coli* housekeeping enzyme (ICD) monitored through targeted proteomics analysis

Enzyme	AGI code	Peptide sequence	Reference
BCAT4	At3g19710	TGEETLAAK	This study
		LYETLSDIQTGR*	This study
		SITNYZPVWIPLAEAK	This study
		GNVVSTPTIAGTILPGVTR	This study
MAM1	At5g23010	SGNASLEEVVMALK	This study
		STYEILSPEDIGIVK	This study
		DGEQSPGGSLTPPQK	This study
		SLGFNDIQFGZEDGGR*	This study
MAM3	At5g23020	GESLMDGVYTK	This study
		ALVVNGAEISSEK	This study
		SGNAPLEEVVMALK	This study
		STYEILSPEDVGIVK*	This study
		DGEQSPGAALTPPQK	This study
IPMS2	At1g74040	GTYEIMSPEEIGLER*	This study
IPMI-LSU1	At4g13430	FILDGEMPSYLQAK*	This study
		VWMDVYALPVPGAGGK	This study
IPMI-SSU3	At3g58990	LGSFALNGLPK	This study
		EDGSSLLINHTTR	This study
		NCVATGEIFPLESEVR*	This study
IPMDH1	At5g14200	LSDAILLGAIGGYK*	This study
		AGSLEGLEFDFK	This study
		IEDAVVDALNK	This study
ICD	JW1122	GPLTTPVGGGIR*	[40]

Peptides marked with asterisk were used for relative quantification.

#### Protein extraction and tryptic digest

Pellet from 1.5 ml culture was harvested by centrifugation and kept at −20°C until extraction. Total protein extraction was performed as previously described [37] and the tryptic digest as described in [38] with the modifications detailed in [39]. Briefly, total protein was obtained by methanol:chloroform extraction, dried in Speed-Vac and dissolved in 100 mM ammonium bicarbonate buffer. Protein concentration was determined by Pierce™ BCA Protein Assay Kit (ThermoFisher, #23225). For tryptic digest, 25–50 μg protein was incubated with 1 μg trypsin/Lys-C mix (Promega, #V5073) over night at 37°C. Digest stopped by acidification with trifluoroacetic acid (TFA) and samples were diluted up to 1.5 ml in buffer containing 2% (v/v) acetonitrile and 0.1% (v/v) formic acid. Peptides were purified over Sep-Pak C-18 columns (Waters, Sep-Pak® Vac 1cc 100 mg, #WAT023590) and eluted in 1 ml buffer containing 65% (v/v) acetonitrile and 0.1% (v/v) formic acid. Purified peptides were dried in Speed-Vac (2–4 h, 1000 rpm, max 35°C). Dried peptides were stored at −20°C until analysis. The dried peptides were resuspended just prior to LC–MS/MS analysis in 25 μl buffer containing 2% (v/v) acetonitrile, 0.5% (v/v) formic acid and 0.1% (v/v) TFA spiked with 20 nM isotopically labelled peptide standards (JPT, SpikeTides™) and filtered through 0.22 μm centrifugal filter (#UFC30GV00, Merck, Darmstadt, Germany).

#### LC–MS analysis

The gradient was adopted from [40,41] with modifications detailed in [39]. Formic acid (0.1% (v/v)) in water and acetonitrile (supplied with 0.1% (v/v) formic acid) were employed as mobile phases A and B, respectively. The elution profile was: 0–1.0 min 5–10% B; 1.0–3.0 min 10–11% B; 3.0–13.0 min 11–19% B; 13.0–21.0 min 19–27.5% B, 21.0–21.7 min 27.5–24% B; 21.7–22.5 min 34–42% B; 22.5–23.5 min 42–90% B, 23.5–26.9 min 90% B, 26.9–30.0 min 90–5% B and 30.0–34.0 min 5% B. Mobile phase flow rate was 500 μl/min and column temperature was maintained at 55°C. Peptide separation was achieved on an Aeris PEPTIDE, XB-C18 column (1.7 μm, 2.1 × 150 mm, Phenomenex, Palo Alto, U.S.A.) on an Advance UHPLC-OLE (Bruker Daltonics, Bremen, Germany). The injection volume was 10 μl.

The following source settings were used for heated electrospray ionisation: spray voltage 3200 V (positive ionisation mode); cone temperature 300°C; cone gas flow 20 psi; heated probe temperature 350°C; probe gas flow 40 and nebuliser gas flow 50. Nitrogen was used as cone and probe gas and argon as collision gas. An EVOQ Elite TripleQuad mass spectrometer (Bruker Daltonics, Bremen, Germany) was scanning for parent ion to fragment ion transitions for individual peptides within scheduled 3 min windows. Resolution of the first and third quadrupole was set to ±1 Da. Detailed information on peptides including retention times, transitions selected for detection and quantification and collision energies can be found in Supplementary Table S3. Skyline 4.2 were used to manually inspect the acquired chromatograms and calculate ratios between endogenous light and synthetic heavy peptides [36]. Relative quantification was obtained by normalising the ratios to the housekeeping protein isocitrate dehydrogenase (ICD). Results were plotted relative to the MAM1 containing strain. Undetected peptides (N/A) were treated as zero values for analysis. Statistical analyses and visualisation were performed in R studio v1.0.153 (R version 3.4.1) [31] using [32,34,35,42].

### Glucosinolate analysis of *A. thaliana* mutants

#### Plant growth and glucosinolate extraction

Seeds of *A. thaliana* ecotype Col-0 (NASC ID N1092), *mam1* [43] and *mam3* (SALK_007222) mutants were cultivated in growth champers with long day light conditions (16 h light, 22°C, 19°C at night, 100–120 μE, 75% relative humidity). Leaf samples were harvested from 3-week-old plants. Material from three plants (approximately 100 mg) were pooled in each sample and three to seven biological replicates were harvested from each genotype. Glucosinolates were extracted and analysed as desulfo-glucosinolates as previously described in [44] with the 96-well optimisation described in [45] and modifications as outlined in [46]. Briefly, one 3 mm chrome ball was added to a microelution tube (Deltalab, cat. no. 408002) with 300 μl 85% methanol containing 10 μM *p*-hydroxybenzyl glucosinolate as internal standard. Immediately after harvest, the leaf tissue was submerged in the extraction solution and the sample was grinded using a mixer mill (3× 30 s at 30 Hz). Samples were spun 10 min at 2500 rpm and the supernatant was loaded on to a 96-well filter plate (MultiScreenHTS, pore size 0.45 μm, EMD Millipore, cat. no. MSHVN 4550) containing 45 μl DEAE-Sephadex A-25 (GE Healthcare, cat. no. 17-0170-02)that had been equilibrated with 300 μl milliQ water. The DEAE-Sephadex columns were washed twice with 70% methanol followed by two washes with milliQ water. The desulfo-glucosinolates were released from the column by over-night incubation with a sulfatase solution. On the following day, the desulfo-glucosinolates were eluted in 100 μl milliQ water and diluted 1:10 in milliQ water prior to LC–MS analysis.

#### LC–MS analysis of glucosinolates

The LC–MS analysis was performed using an Advance UHPLC system (Bruker, Bremen, Germany) with a Kinetex XB-C18 column (100 × 2.1 mm, 1.7 μm, Phenomenex, Palo Alto, U.S.A.) coupled to an EVOQ Elite TripleQuad mass spectrometer equipped with an electrospray ionisation source (ESI) (Bruker, Bremen, Germany). The method was performed as previously described [46,47] using formic acid (0.05%) in water and formic acid (0.05%) in acetonitrile as mobile phase A and B, respectively. Ionisation was obtained by electrospray ionisation in positive ionisation mode with spray voltage of 3500 V and probe and cone temperatures at 400 and 350°C, respectively. Parent ion to product ion transitions were monitored for one aromatic (2-phenylethyl), three indolic (indol-3-ylmethyl, *N*-methoxy-indol-3-ylmethyl, 4-methoxy-indol-3-ylmethyl) and nine aliphatic (3-methylthiopropyl, 3-methylsulfinylpropyl, 4-methylthiobutyl, 4-methylsulfinylbutyl, 5-methylsulfinylpentyl, 7-methylthioheptyl, 7-methylsulfinylheptyl, 8-methylthiooctyl, 8-methylsulfinyloctyl) glucosinolates. Quantification of individual glucosinolates was done using *p*-hydroxybenzyl glucosinolate as internal reference. Further details and transitions can be found in [46,47].

## Results

### Protein structure modelling and phylogeny analysis of MAM sequences

**Figure 3:**
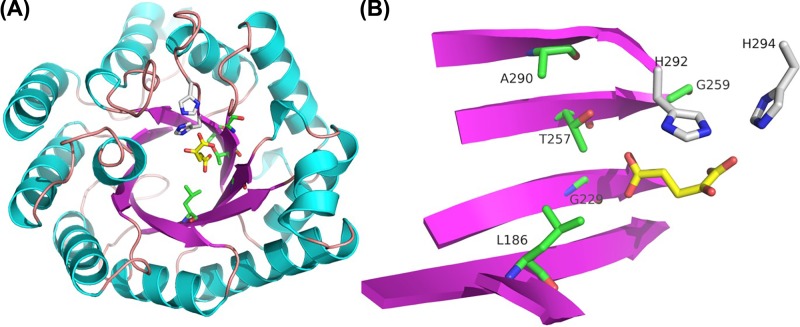
Homology model of MAM1 from *A. thaliana* (**A**) The model of MAM1 was based on the crystal structure of *N. meningitis* IPMS (PDB-ID 3RMJ). β sheets (purple) and α helixes (turquoise) comprise the (β/α)_8_ catalytic barrel characteristic of IPMS/MAM family. The binding of IPMS substrate is shown in yellow. Green residues represent the five residues in MAM1 predicted to be within 8 Å of the substrate. Grey residues represent two His residues conserved in the IPMS/MAM family. (**B**) Simplified presentation of the residues in MAM1, which is predicted to be in close proximity of the substrate. Colours as in (A).

From a protein sequence alignment of MAM1, MAM3, IPMS1 and IPMS2 from *A. thaliana* ecotype Col-0 and MAM2 from ecotype Ler-0 polymorphic residues specific to MAMs were identified. To date, no MAM enzyme has been crystallised. Since IPMS and MAM enzymes essentially perform the same reaction, we chose to homology model the structure of MAM1 based on a crystallised IPMS (PDB-ID 3RMJ) (Figure 3A). Homocitrate synthases (HCS) are also of the DRE-TIM metallolyase superfamily. Assuming the MAM substrate binds in a similar fashion as in HCS, we retrieved a crystallised HCS (PDB-ID 2ZTJ [48] from *Thermus thermophilus* (*Tt*HCS) with α-ketoglutarate bound in the catalytic pocket. We superimposed the MAM1 homology model on to 2ZTJ (RMSD of 1.3 Å over 202 residues) and selected MAM1 residues within 8 Å distance of α-ketoglutarate (Supplementary Figure S5). This subset of residues was used to predict four polymorphic residues (Figure 3B). Three of these sites were previously described as interacting with the substrate (Leu186, Thr257 and Gly259). The remaining residue (Ala290) was included as it fulfilled the criteria of distance to substrate and polymorphism. A fifth residue (Gly229) was within 8 Å of the substrate, but remained constant for all MAM synthases and was thus not targeted for mutation.

A phylogenetic analysis including 57 MAM synthase protein sequences representing 12 species from lineages I and II in the Brassicaceae family was performed. Lineage III is not represented, as to the best of our knowledge no species has been sequenced. This analysis was used to design the substitution mutations so that the mutants represented the natural variation observed at these positions (Table 2 and Supplementary Figure S1). Fifteen substitutions were chosen and residues were mutated in the *A. thaliana* MAM1. In addition, three mutants were constructed, which carried two or three mutations in combination. These were inspired from the MAMb clade, which includes MAM3 variants. In this clade, the T257G and G259A mutations are common, and these were therefore combined into the double mutant, GA. Two triple mutants were constructed to mimic specifically MAM3s of *A. thaliana* and *C. sativa*, which are both able to elongate methionine with more than three methylene groups. The mutants were named GAM (T257G/G259A/L186M) and GAG (T257G/G259A/A290G), respectively.

**Table 2:** Polymorphic residues in selected MAM synthases (numbering according to *A. thaliana* MAM1)

Natural MAM variant	186	257	259	290
AtIPMS	H	N	P	S
AtMAM1	L	T	G	A
AtMAM2	L	N	A	S
AtMAM3	M	G	A	A
AtbMAM1	L	S	G	A
Al/AcMAMb	I/L	G	A	T
Cs/CrMAM3	T	G	A	A
Al/AcMAM3/c	C	C	A	S
Cs/CrMAM3	G/I	G	A	G
NA	–	V	–	–
NA	–	D	–	–

Selected examples of natural variance in the residues targeted for mutation is shown. Species are abbreviated as follows: Ac, *A. cebennensis*; Al, *A. lyrata*; At, *A. thaliana*; Atb: *A. thaliana* Br-o; Cr, *C. rubella*; Cs, *C. sativa*; NA, not found in our analysis but chosen for structural characteristics.

### Metabolite analysis of *E. coli* expressing the chain elongation pathway with MAM variants

#### Protein amino acid pools in *E. coli* expressing the chain elongation pathway

**Figure 4:**
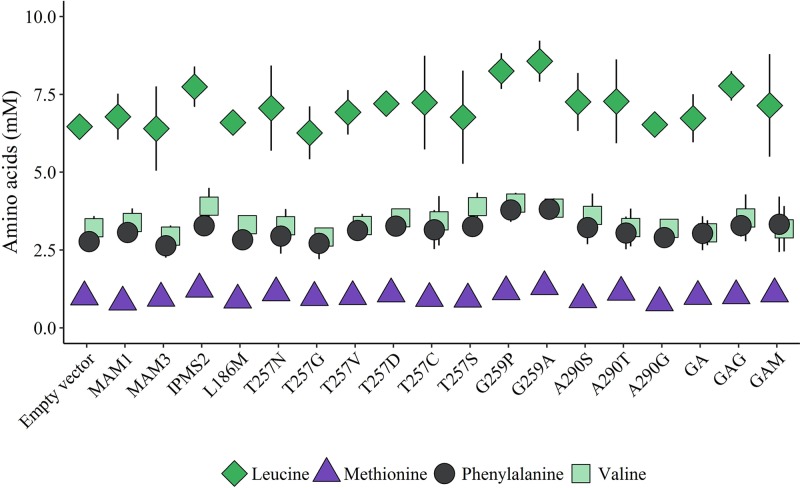
Levels of protein amino acid substrates in *E. coli* expressing the chain elongation pathway with different MAM variants GA, GAG and GAM represent MAM variants with multiple mutations; GA, T257G/G259A; GAG, T257G/G259A/A290G; GAM, T257G/G259A/L186M. Levels of leucine, phenylalanine and valine are statistically unchanged between the constructs when comparing to the average mean of all cultures. Methionine levels vary between cultures, although the differences are small. Data represent average and standard deviation of three biological replicates each grown in three technical replicates.

**Figure 5:**
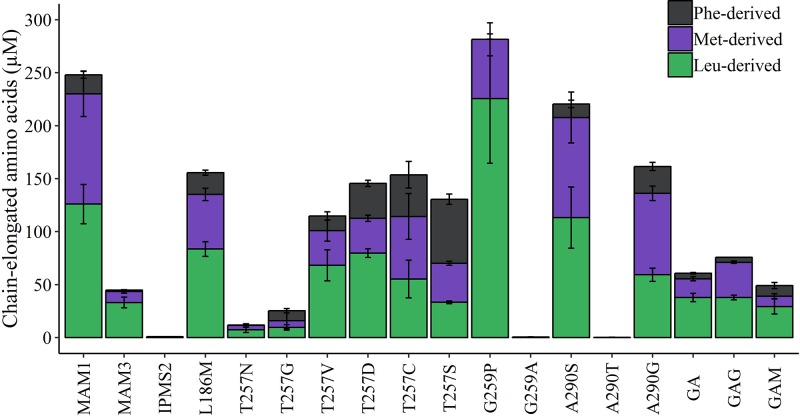
Production of chain-elongated products in all MAM variants Products are grouped according to precursor amino acid: methionine (purple), phenylalanine (grey) and leucine (green). Methionine-derived products include methionine elongated from one to six cycles, leucine-derived products include leucine elongated once or twice and phenylalanine-derived product is only elongated once. Table shows the percentage of total production within each group of compounds. Data represent average and standard deviation of three biological replicates each grown in three technical replicates.

Amino acids used as precursors for the chain elongation pathway based on previous data from mutant plants [49] and heterologous host organisms [10,20] are leucine, methionine, phenylalanine and valine. In the *E. coli* strains expressing the chain elongation pathway with different variants of the MAM synthases, protein amino acid levels were monitored and compared with the levels of *E. coli* strain transformed with empty vector (Figure 4 and Supplementary Figure S6). Generally, the cultures contained approximately three times more phenylalanine than methionine and seven times more leucine than methionine. The levels of leucine, phenylalanine and valine did not statistically differ from the overall mean of all cultures. Methionine levels showed small, but statistically significant differences between cultures (Supplementary Table S4). Slightly reduced levels were seen in the *E. coli* strains producing high levels of methionine-derived products (Figures 4 and 5). However, this did not affect cell density, and thus is likely an effect of the methionine pool being smaller than those of phenylalanine and leucine.

#### Levels of chain-elongated amino acids produced by *E. coli*

By LC–MS analysis of the medium in cultures of *E. coli* expressing the chain elongation pathway with different MAM variants, we detected nine elongated amino acids derived from one to six elongation cycles: l-homomethionine (HM), l-dihomomethionine (DHM), l-trihomomethionine (TriHM), l-tetrahomomethionine (TetraHM), l-pentahomomethionine (PentaHM), l-hexahomomethionine (HexaHM), l-homoleucine (HL), l-dihomoleucine (DHL), and l-homophenylalanine (HPhe). Additionally two were monitored but not detected: l-trihomoleucine (TriHL) and l-heptahomomethionine (HeptaHM). Strains harbouring the constructs containing the native MAM1 enzyme produced nearly 5-fold more elongated products than those containing MAM3. In strains expressing the G259P variant, we observed an increase in leucine-derived products to 80% of the total products. This was accompanied with a decrease in methionine-derived products and an elimination of HPhe production. In 7 out of 13 strains expressing functional MAM1 variants, the percentage of leucine-derived products increased, while the level of methionine-derived products decreased compared with MAM1. However, only in two of these seven strains did HPhe production decrease, while an increase was seen in the remaining five strains. Similarly, HPhe level was increased in all strains expressing MAM1 variants with lowered levels of leucine-derived products. Two strains – A290S and GAG – showed unchanged composition of chain-elongated amino acids, although the production in GAG was dramatically reduced. Strains harbouring constructs with IPMS2 or the two MAM1 variants (G259A and A290T) did not produce any of the chain-elongated amino acid products (Figure 5). The composition of chain-elongated amino acids produced by the MAM1 variants indicates that the targeted substitutions have changed the substrate specificity of almost all of them. Overall, MAMa homologues and MAM1 variants mimicking MAMa produced higher levels than either MAMb homologues, MAM1 variants mimicking MAMb or IPMS enzymes (Figure 5).

**Figure 6:**
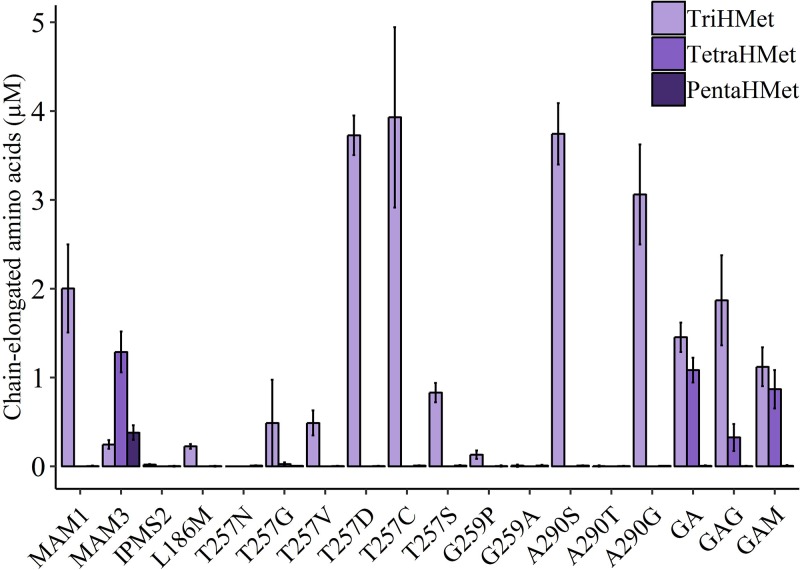
Production of amino acids elongated with more than two methylene groups MAM3 is the only MAM variant that produces five and six times elongated products. HexaHM levels are below the limit of quantification and thus not included in the graph. Data represent average and standard deviation of three biological replicates each grown in three technical replicates.

The number of recursive cycles is affected by the mutations in MAM1 (Figure 6 and Supplementary Figure S7). The native MAM1 enzyme produces predominantly methionine- and leucine-derived products that have been elongated once or twice and low levels of the thrice-elongated methionine (TriHM). This is consistent with previously published results [20]. MAM3 produces a larger variety of products – mostly of once-elongated products (HM and HL), almost no thrice-elongated products (TriHM and TriHL), and substantial amounts of both four and five times elongated methionine (Tetra- and PentaHM). In addition, the six times elongated methionine (HexaHM) was detected below the limits of quantification. Four single mutation MAM1 variants (T257D/C, A290S/G) produced more TriHM than the native MAM1. The GA, GAG and GAM variants produced TetraHM. These were the only variants besides MAM3 that were able to generate products elongated more than three times. Interestingly, MAM1 variants carrying the single mutation (either T257G or G259A) – that are combined in the GA double variant – did not produce amino acids elongated more than twice. Thus, reducing size of the residue Thr257 alone does not allow for bigger substrate – on the contrary, it reduces the number of cycles. When the T257G substitution was combined with a larger residue on Gly259, the ability to elongate methionine three times was restored and even increased to four rounds. This suggests that the binding or interaction with the substrate might have been compromised in the single mutations, but restored when combining them.

### Analysis of *Arabidopsis mam1* and *mam3* mutants for HPhe-derived glucosinolate

**Figure 7:**
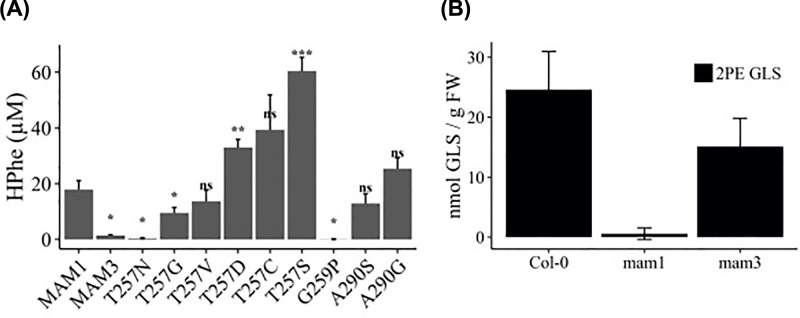
Production of HPhe in E. coli by MAM1 variants and the effect of MAM mutants on HPhe-derived glucosinolates in A. thaliana (**A**) Production of HPhe in MAM variants with changed specificity toward phenylalanine. Data represent average and standard deviation of three biological replicates each grown in three technical replicates. Tukey’s HSD (Honestly Significant Difference) was used for statistical analysis. Variants marked with asterisk denotes significant changes: *P*-values: 0.01–0.05 = *; *P*-values: 0.001–0.01 = **; *P*-values: 0–0.001 = ***. (**B**) Glucosinolate analysis in *A. thaliana* ecotype Col-0 and knockout mutants *mam1* and *mam3*. Data represent average and standard deviation of 3–7 biological replicates.

Noticeably, both MAM1 and MAM3 were able to produce HPhe, with MAM1 having nearly 20-fold higher levels than MAM3 (Figure 7A). The HPhe levels were affected by mutations in the residues Thr257 and Gly259. Dependent on the substitution, mutations in residue Thr257 can either decrease or increase specificity towards phenylalanine, with T257S showing nearly 3-fold higher HPhe production than the native MAM1. Additionally, mutating Gly259 eliminates production of HPhe. HPhe production in functional variants with mutations in Leu186 and Ala290 were similar to that of MAM1.

The results were contradicting with the previously proposed role of MAM3 [10], but could potentially be an effect of expressing the enzymes in a heterologous system. To investigate this further and determine the role of MAM1 and MAM3 in production of the HPhe-derived 2-phenylethyl glucosinolate *in planta*, we performed glucosinolate analysis on leaves of *mam1* (TU1 [43] and *mam3* (SALK_007222) mutants. 2-Phenylethyl glucosinolate was absent from the *mam1* and present in the *mam3* mutant, thus demonstrating that MAM1 is responsible for production of this glucosinolate in *A. thaliana* (Figure 7B and Supplementary Figure S8).

### Protein expression assessed by targeted proteomics

**Figure 8:**
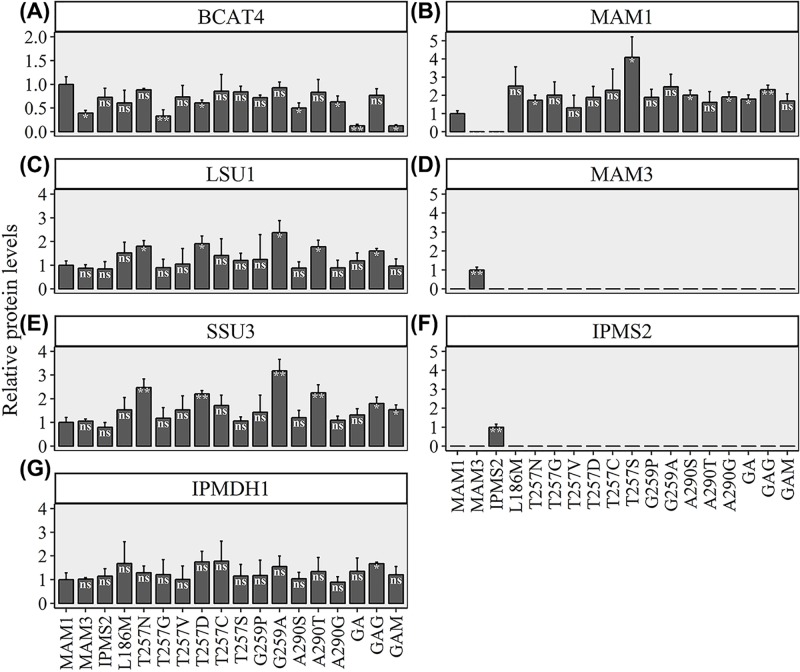
Protein levels in *E. coli* strains expressing the chain elongation pathway genes (**A**) BCAT4, (**B**) MAM1, (**C**) LSU1, (**D**) MAM3, (**E**) SSU3, (**F**) IPMS2 and (**G**) IPMDH1. A representative peptide for each protein was used for relative quantification. Data represent average and standard deviation of three biological replicates.

Protein levels of the chain elongation enzymes expressed in *E. coli* were monitored by targeted proteomics of proteotypic peptides representing the individual proteins. Seven proteins were monitored: MAM1, MAM3, IPMS2, BCAT4, LSU1, SSU3 and IPMDH1 (Figure 8 and Supplementary Figure S9). LSU1 and IPMDH1 were present at similar levels in all strains (Figure 8C,D), while both BCAT4 and SSU3 had altered expression in some strains. In strains harbouring MAM3 or the MAM1 variants T257G, GA and GAM, BCAT4 was lower than in the MAM1-containing strain (Figure 8A). In strains harbouring the MAM1 variants T257N, T257D, G259A and A290T, SSU3 protein levels were higher than in the strain harbouring MAM1 (Figure 8E). No chain-elongated amino acid products were found in strains expressing G259A and A290T and only low levels in T257N (Figure 5). However, the T257D had good production levels. Thus, higher SSU3 levels do not block chain elongation, and not all constructs resulting in low levels or no chain-elongated products have elevated SSU3 levels, as evident from the analysis of IPMS2 and T257G.

Regarding the protein levels of MAM1 variants, only one construct resulted in changed levels compared to the native MAM1 (Figure 8A). T257S showed higher MAM1 levels, although this did not result in correspondingly higher levels of chain-elongated products (Figure 5). As these results represent a relative quantification of protein levels, MAM3 (Figure 8D) and IPMS2 (Figure 8F) cannot be directly compared with the MAM1 variants. The approach, however, allows us to confirm expression of all biosynthetic enzymes.

## Discussion

We set out to obtain a better understanding of MAM1 substrate specificity and product profile, and thereby the number of recursive cycles of the chain elongation pathway in glucosinolate biosynthesis. Amino acid residues in MAM1 were selected for mutagenesis based on MAM-specific polymorphic residues identified in a phylogenetic analysis of 57 MAM protein sequences from 12 Brassicaceae species as well as the distance of these to the substrate. The latter was estimated from a MAM1 homology model and a TtHCS structure co-crystallised with a substrate [48]. The natural variation seen in MAM-specific polymorphic residues were mimicked in the MAM1 variants generated in the present study. Three of the residues mutated in MAM1 based on our selection criteria were predicted previously to be interacting with the substrate: two in the active site (Thr257 and Gly259) and one in the substrate pocket (Leu186) [12]. This validated our approach and selection criteria for identification of potentially important residues. We identified a fourth residue (Ala290) that was included in the analysis.

We monitored the effect of protein engineering MAM1 by analysing the level and profile of chain-elongated amino acid products in medium of *E. coli* strains expressing the chain elongation pathway. As the Thr257 residue was highly polymorphic, it is represented by more substitution mutations than the other targeted residues in the library of MAM1 variants. The lowest producing strain harboured the T257N MAM1 variant, which suggests that introducing an amide group at the bottom of the active site may result in sterical hindrance, thereby lowering activity of the enzyme. The T257G variant produced severely reduced levels of elongated products compared with other MAM1 variants as well as MAM3. When the T257G substitution was performed in *Mt*IPMS it caused loss of activity in *E. coli* [12], which is in agreement with our results. MAM1 variants with substitutions T257V, T257D, 257C and T257S all produced a similar range of total products, but with different profiles of chain-elongated amino acids (Figure 5). T257S had an almost 3-fold increase in HPhe production compared with MAM1 (Figure 7A) and produced more phenylalanine-derived products than leucine- and methionine-derived products combined. The T257C substitution mimics MAMc, which is found in e.g. *C. sativa*, which produces glucosinolates derived from methionine derivatives from nine cycles of chain elongation [50]. Together with T257D, A290G and A290S, T257C had the highest TriHM production amongst the tested MAM1 variants including native MAM synthases. The production of TriHM as well as DHM in the T257C MAM1 variant confirms that homologues of MAMc have evolved to be iterative enzymes.

The IPMS2 mimicking G259P variant produced more chain-elongated leucine than methionine products. The increase in leucine-derived products was almost 2-fold. It was also the only MAM variant unable to elongate phenylalanine. The substitution has replaced glycine with the much bulkier proline. Such a substitution would normally be expected to reduce activity of an enzyme. However, proline can also introduce a bend in the β-sheet, which in this case could make room for the methylene branch on the leucine side chain [51].

The MAM1 variants G259A and A290T and IPMS2 did not generate chain-elongated amino acid products in *E. coli*, but the presence of the proteins was confirmed by targeted proteomics. The IPMS2 enzyme only elongated valine, and thus was not expected to produce other products. When the A290T mutation is introduced into MAM1 of *A. thaliana*, it generates a *mam1* knockout phenotype [9], which is in agreement with our data.

In *A. thaliana*, glucosinolates derived from chain-elongated leucine products are only seen in mutants that are altered in their branched chain amino acid metabolism [49,52]. However, when MAM1 variants are expressed together with the chain elongation enzymes in *E. coli*, chain-elongated leucine products were produced by all the *E. coli* strains. This could be explained by a loss of specificity when moving enzymes to heterologous hosts, but it could also suggests that leucine biosynthesis and methionine chain elongation are physical separated at the cellular level in the native host *A. thaliana*. Such model was previously proposed [53] and would allow for a localised high methionine-to-leucine ratio and thus hinder by-product formation. Indeed, feeding MAM1 with methionine reduced the proportion of leucine-derived by-products [20]. However, such physical separation into distinct localisations of the respective enzymes at the cellular level has yet to be proven.

We provide conclusive *in planta* evidence that MAM1 and not MAM3 as hitherto proposed is responsible for production of the HPhe-derived 2-phenylethyl glucosinolate (Figure 7A). The data are in agreement with the observation that the only MAM present in *Barbarea vulgaris* that produces high levels of 2-phenylethyl glucosinolate is a MAM1 homologue [54].

## Supporting information

**Supplementary Figure S1:** Alignment of 57 MAM and 2 IPMS full protein sequences. According to *A. thaliana* MAM1 numbering, only Leu186, Thr257, Ile258, Gly259 and Ala290 residues are shown.

**Supplementary Figure S2:** Matrix from the alignment of MAM and IPMS enzyme sequences. Upper part indicates sequence similarity in percentage and the lower part shows differences between the protein sequences. Two MAM sequences have been removed from the matrix, as the comparison indicated low sequence similarity due to poor quality of the sequence data.

**Supplementary Figure S3:** LC-MS traces of homomethionine (HM), dihomomethionine (DHM), trihomomethionine (TriHM), tetrahomomethionine (TetraHM), pentahomomethionine (PentaHM) and hexahomomethionine (HexaHM). A representative sample of MAM1 and MAM3 containing strains as well as an empty vector control are shown. For each compound, the pure sample is shown in the top panel.

**Supplementary Figure S4:** LC-MS traces of heptahomomethionine (HeptaHM), homoleucine (HL) and homophenylalanine (HPhe). A representative sample of MAM1 and MAM3 containing strains as well as an empty vector control are shown. For each compound, the pure sample is shown in the top panel.

**Supplementary Figure S5:** Homology model of MAM1 from *A. thaliana.* (yellow) superimposed on the crystal structure of *N. meningitis* IPMS (green). Only the (β/α)_8_ catalytic barrel is shown. The RMSD value is 0.26 Å.

**Supplementary Figure S6:** Protein amino acids monitored in this study. Each graph shows levels of one amino acid in the media of *E. coli* strains expressing MAM variants and an empty vector control. The mean of all cultures is indicated by the red line. ANOVA was used to investigate changes in the individual amino acids between the cultures. p-values are shown in upper right corner of each graph. Data represents average and standard deviation of three biological replicates each grown in three technical replicates.

**Supplementary Figure S7:** Produced chain elongated amino acids were grouped according to how many methylene groups were added to the methionine, phenylalanine or leucine structures (one, two or three and above). Bars represents the percentage of each group of products relative to the total production within each culture. Data represents average and standard deviation of three biological replicates each grown in three technical replicates.

**Supplementary Figure S8:** Glucosinolate analysis in *A. thaliana* ecotype Col-0 and knockout mutants mam1 and mam3. Glucosinolates (GLS) were grouped into aromatic (2PE) GLSs, indole GLSs and short chain (SC - one to three elongation rounds) and long chain (LC - four elongation rounds and above) aliphatic GLSs. Data represents average and standard deviation of 3-7 biological replicates.

## Abbreviations

BCAT4: branched-chain aminotransferase 4
DHL: dihomoleucine
DHM: dihomomethionine
HCS: homocitrate synthase
HeptaHM: heptahomomethionine
HexaHM: hexahomomethionine
HL: homoleucine
HM: homomethionine
HPhe: homophenylalanine
ICD: isocitrate dehydrogenase
IPMDH: isopropylmalate dehydrogenase
IPMI: isopropylmalate isomerase
IPMS: isopropylmalate synthase
LSU1: IPMI large subunit 1
MAM: methylthioalkylmalate synthase
PentaHM: pentahomomethionine
SSU3: IPMI small subunit 3
TetraHM: tetrahomomethionine
TFA: trifluoroacetic acid
TriHL: trihomoleucine
TriHM: trihomomethionine
