# Supplementary material for: Changing substrate specificity and iteration of amino acid chain elongation in glucosinolate biosynthesis through targeted mutagenesis of Arabidopsis methylthioalkylmalate synthase 1
Source: Biosci Rep. 2019 Jul 2;39(7):BSR20190446. doi: 10.1042/BSR20190446 (PMC6603273; doi:10.1042/BSR20190446)
Supplement: Supplementary file 1 [file BSR20190446_Supp1.pdf]

Arabidopsis thaliana Col-0 (NP_001330047.1) AtMAM1	LTIGA	5
Arabidopsis thaliana mt-0 (AJ486884.1) AtmMAM1	LTIGA	5
Arabidopsis thaliana sorbo (CAD31141.1) AtsMAM1	LTIGA	5
Arabidopsis thaliana Br-0 (1001 genomes 6904) AtbMAM1	LSIGA	5
Brassica oleracea var. oleracea (XP_013618041.1) BooMAM1.1	VACPS	5
Brassica napus (XP_013678219.1) BnMAM1.1	VACPS	5
Brassica rapa (XP_009130133.1) BrMAM1.1	VACPS	5
Eruca vesicaria subsp. sativa (AGS49163.1) EvsMAM	LACPS	5
Arabidopsis thaliana Col-0 (NP_173285.2) AtIPMS1	HNIPS	5
Arabidopsis thaliana Col-0 (NP_177544.1) AtIPMS2	HNIPS	5
Brassica napus (XM_013817468.2) BnMAM1.2	INIGS	5
Brassica rapa (XM_009138151.2) BrMAM1.2	INIGS	5
Brassica rapa subsp. pekinensis (ACR10246.1) BrpMAM.1	INIGS	5
Raphanus sativus (XM_018606131.1) RsMAM1.2	LNLGS	5
Arabidopsis thaliana Ler-0 (Q8VX04.1) AtMAM2	LNVAS	5
Arabidopsis cebennensis (CAJ55509.1) AcMAMa	LNVAS	5
Arabidopsis lyrata lyrata (XP_002872029.1) AllMAM2	LNVAS	5
Arabidopsis lyrata petraea (CAJ55506.1) AlpMAMa	LNVAS	5
Eutrema salsugineum (XP_006394566.2) EsMAM2	LNVAS	5
Camelina sativa (XP_010493409.1) CsMAM2.1	LNVAS	5
Camelina sativa (XP_010421098.1) CsMAM2.2	LNVAS	5
Camelina sativa (XP_010454580.1) CsMAM1	LNVAS	5
Boechera divaricarpa (CAJ55512.1) BdMAMa	LNVAS	5
Capsella rubella (XP_006289820.1) CrMAM2	LNVAS	5
Brassica oleracea var. oleracea (XP_013593685.1) BooMAM2	LNVAS	5
Brassica napus (XP_013730921.2) BnMAM2	LNVAS	5
Raphanus sativus (XP_018477323.1) RsMAM2	LNVAS	5
Eutrema salsugineum (XP_006394567.1) EsMAM1	LNIAS	5
Brassica rapa (XM_009138149.2) BrMAM1.4	LNLAS	5
Brassica rapa subsp. pekinensis (ACR10248.1) BrpMAM.2	LNLAS	5
Brassica oleracea var. oleracea (XM_013763569.1) BooMAM1.2	FTLAS	5
Brassica rapa (XM_009146283.2) BrMAM1.3	FTLAS	5
Raphanus sativus (XP_018457526.1) RsMAM1.1	FTLAS	5
Arabidopsis lyrata lyrata (CAJ55517.1) AllMAMc.1	CCIAS	5
Arabidopsis lyrata subsp. lyrata (XP_020879218.1) AllMAM3.2	CCIAS	5
Arabidopsis lyrata subsp. lyrata (EFH48290.1) AllMAMc.2	CCIAS	5
Arabidopsis lyrata petraea (CAJ55508.1) AlpMAMc	CCIAS	5
Camelina sativa (XP_019083909.1) CsMAM3.3	CCIAS	5
Capsella rubella (XP_006289994.1) CrMAM1	CCIAS	5
Arabidopsis cebennensis (CAJ55511.1) AcMAMc	CCIAS	5
Boechera divaricarpa (CAJ55514.1) BdMAMc	CCIAS	5
Camelina sativa (XP_010493411.1) CsMAM3.1	CCIAS	5
Camelina sativa (XP_010454582.1) CsMAM3.7	CCIAS	5
Capsella rubella (XM_006289411.2) CrMAM3.2	CCVAG	5
Camelina sativa (XM_010473736.2) CsMAM3.4	GGFAG	5
Camelina sativa (XM_010418504.2) CsMAM3.5	GGFAG	5
Capsella rubella (XM_006301203.2) CrMAM3.3	IGFAG	5
Camelina sativa (XM_010430647.2) CsMAM3.6	IGFAG	5
Boechera divaricarpa (CAJ55513.1) BdMAMb	IGFAA	5
Arabidopsis lyrata lyrata (XP_002872030.1) AllMAM3.1	LGFAA	5
Arabidopsis lyrata subsp. petraea (CAJ55507.1) AlpMAMb	LGFAT	5
Arabidopsis cebennensis (CAJ55510.1) AcMAMb	IGFAT	5
Arabidopsis thaliana Col-0 (NP_197693.1) AtMAM3	MGFAA	5
Arabidopsis thaliana Br-0 (1001 genomes 6904) AtbMAM3	MGFAA	5
Arabidopsis thaliana Ler-0 (1001 genomes 7213) AtlMAM3	MGFAA	5
Arabidopsis thaliana sorbo (AJ486943.1) AtsMAM3	MGFAA	5
Camelina sativa (XP_010421099.1) CsMAM3.2	TGFAA	5
Camelina sativa (XP_010493410.1) CsMAM3.8	TGFAA	5
Capsella rubella (XP_006290200.1) CrMAM3.1	TGFAA	5

		1	2	3	4	5	6	7	8	9	10	11	12	13	14	15	16	17	18	19	20	21	22	23	24	25	26	27	28	29	30	31	32	33	34	35	36	37	38	39	40	41	42	43	44	45	46	47	48	49	50	51	52	53	54	55	56	57
AtMAM1	1		100,00	99,41	95,45	92,11	92,11	91,32	85,85	86,05	82,09	87,82	85,32	75,55	79,49	79,53	79,96	79,61	79,84	79,65	73,59	79,06	75,73	77,60	77,80	77,21	77,80	79,02	77,76	75,49	76,67	73,92	75,98	69,85	77,56	77,56	76,97	77,17	76,97	79,33	75,59	77,17	75,83	72,05	71,65	70,67	71,71	72,62	72,82	65,94	60,89	60,95	60,75	76,32	75,93	76,22	45,31	44,70
AtmMAM1	2	0		99,41	95,45	92,11	92,11	91,32	85,85	86,05	82,09	87,82	85,32	75,55	79,49	79,53	79,96	79,61	79,84	79,65	73,59	79,06	75,73	77,60	77,80	77,21	77,80	79,02	77,76	75,49	76,67	73,92	75,98	69,85	77,56	77,56	76,97	77,17	76,97	79,33	75,59	77,17	75,83	72,05	71,65	70,67	71,71	72,62	72,82	65,94	60,89	60,95	60,75	76,32	75,93	76,22	45,31	44,70
AtsMAM1	3	3	3		95,26	91,91	91,91	91,12	86,05	86,05	81,89	87,82	85,32	75,55	79,49	79,53	80,15	79,61	80,04	79,84	73,77	79,26	75,73	78,00	78,19	77,60	78,19	79,02	78,15	75,69	76,86	74,12	76,17	70,22	77,76	77,76	77,17	77,36	77,17	79,53	75,79	77,36	75,83	72,24	71,85	70,87	72,10	72,62	72,82	65,94	60,89	60,95	60,75	76,52	76,13	76,41	45,31	44,70
AtMAM2	4	23	23	24		93,69	93,69	92,90	87,03	87,23	82,87	88,41	86,69	76,65	80,66	80,12	81,09	79,41	79,26	79,06	73,22	78,47	74,95	78,59	78,78	78,19	78,78	80,00	78,74	75,10	76,47	73,92	75,59	69,85	75,79	75,98	75,20	74,61	74,41	77,56	74,41	75,79	74,66	71,06	70,67	70,47	71,51	71,46	71,65	65,35	61,28	61,54	61,34	75,34	74,95	75,24	44,69	43,93
AllMAM2	5	40	40	41	32		99,21	96,84	88,80	88,61	84,84	90,37	88,45	77,57	81,45	81,73	82,06	80,63	78,91	78,71	72,73	77,73	74,41	78,04	78,24	77,65	78,24	80,43	77,80	74,76	76,52	73,78	75,44	69,54	76,62	76,82	76,03	75,64	75,44	78,19	73,48	75,05	74,31	69,94	69,16	68,96	70,59	71,51	71,71	65,03	60,97	61,22	61,02	75,39	75,00	75,68	45,00	44,55
AlpMAMa	6	40	40	41	32	4		96,84	89,00	88,80	85,04	90,37	88,65	77,76	81,64	81,93	82,24	80,23	79,10	78,91	72,91	77,93	74,61	78,04	78,24	77,65	78,24	80,43	77,80	74,56	76,32	73,58	75,24	69,54	76,62	76,82	76,03	75,64	75,44	78,39	73,48	75,05	74,31	69,94	69,16	68,96	70,78	71,51	71,71	65,03	60,97	61,42	61,22	75,00	74,61	75,49	45,16	44,70
AcMAMa	7	44	44	45	36	16	16		88,02	87,82	83,86	89,39	87,87	76,84	80,66	80,75	81,31	79,84	78,12	77,93	71,82	76,76	74,22	77,45	77,65	77,06	77,65	80,23	77,41	74,56	75,93	73,19	75,44	69,54	75,83	76,03	75,25	74,85	74,66	77,60	73,08	74,66	74,12	69,55	68,76	68,57	70,59	71,32	71,51	64,83	61,17	60,83	60,63	74,22	73,83	74,90	44,69	44,24
CsMAM2.1	8	72	72	71	66	57	56	61		97,42	92,06	93,45	92,13	77,39	81,45	80,94	80,93	78,54	78,52	78,32	72,18	77,15	73,63	76,67	76,86	76,27	76,86	76,91	77,67	73,39	75,34	71,82	74,07	69,17	75,25	75,44	75,64	75,83	76,03	76,62	72,69	73,92	72,94	69,17	68,38	68,18	70,61	71,07	71,26	64,82	59,88	60,79	60,79	73,87	73,28	74,07	45,62	44,55
CsMAM2.2	9	71	71	71	65	58	57	62	13		92,86	93,85	92,91	77,39	81,45	80,94	80,75	78,74	79,10	78,91	73,09	77,73	73,63	77,45	77,65	77,06	77,65	77,69	78,46	73,39	75,54	72,21	74,66	69,54	74,85	75,05	75,64	76,03	76,23	77,41	72,89	74,12	73,53	69,17	68,38	68,18	70,41	71,65	71,84	65,42	60,67	61,39	61,39	74,07	73,48	74,46	45,62	44,55
CsMAM1	10	91	91	92	87	77	76	82	40	36		89,09	87,99	73,53	77,34	77,01	77,20	78,31	75,64	75,44	69,29	74,07	75,56	73,14	73,33	72,75	73,33	72,99	73,91	70,45	72,02	68,88	72,02	66,79	72,10	72,30	72,69	73,08	73,28	74,26	69,74	70,78	70,00	66,60	65,81	66,00	67,86	70,75	70,75	65,11	59,96	60,20	60,20	69,74	69,16	70,57	44,20	43,73
BdMAMa	11	62	62	62	59	49	49	54	33	31	55		92,32	78,12	82,23	82,51	81,87	80,31	80,27	80,08	73,82	78,91	75,20	78,82	79,02	78,43	79,02	78,86	79,05	74,17	76,32	73,19	75,24	70,09	75,83	76,03	76,62	76,42	76,23	78,59	73,87	74,90	74,12	69,96	69,17	68,77	71,20	72,43	72,62	66,21	61,84	61,98	61,78	75,05	74,26	74,66	45,78	45,17
CrMAM2	12	75	75	75	68	59	58	62	40	36	61	39		76,56	80,54	79,84	79,70	78,47	78,40	78,21	72,10	76,85	73,35	76,37	76,56	75,98	76,56	76,61	77,95	72,51	74,46	72,32	73,98	69,29	75,15	75,34	75,73	75,34	75,34	77,10	72,02	72,99	72,66	69,09	68,31	68,11	70,53	70,66	70,85	64,44	59,92	60,43	60,24	72,21	71,62	72,82	45,48	44,57
BooMAM2	13	133	133	133	127	122	121	126	123	123	144	119	128		93,15	87,22	87,82	75,46	75,41	75,23	74,95	75,41	71,01	72,79	72,98	72,43	73,35	72,66	72,56	69,72	70,83	67,71	70,28	69,33	72,19	72,38	73,11	72,19	72,01	73,11	70,53	71,32	71,14	64,94	64,21	63,84	65,19	68,49	68,67	62,92	57,95	58,04	57,86	68,50	68,13	69,53	43,60	42,88
BnMAM2	14	105	105	105	99	95	94	99	95	95	116	91	100	37		91,73	84,89	79,18	79,34	79,14	73,14	79,34	74,66	76,56	76,76	76,17	77,15	76,41	76,32	73,29	74,46	71,15	73,59	68,37	75,93	76,13	77,10	75,93	75,73	76,91	74,17	75,00	74,80	68,24	67,45	67,06	68,69	72,20	72,39	66,67	61,24	61,49	61,30	71,98	71,60	73,06	45,16	44,39
RsMAM2	15	104	104	104	101	93	92	98	97	97	117	89	103	69	42		84,40	79,22	79,96	79,76	73,31	79,96	75,05	76,77	76,97	76,38	77,17	76,62	76,68	73,67	74,85	72,30	73,97	68,88	76,13	76,33	77,32	75,94	75,74	76,53	74,36	75,59	75,00	68,32	67,52	66,93	68,77	71,65	71,84	65,74	60,67	61,90	61,71	71,96	71,57	73,83	45,31	45,17
EsMAM2	16	107	107	106	101	96	95	100	102	103	122	97	109	66	81	83		80,41	79,44	79,25	77,88	79,25	74,39	76,59	76,78	76,22	76,97	76,45	75,80	72,52	73,64	70,65	72,90	70,02	75,23	75,42	75,23	74,67	74,48	75,98	72,05	72,66	72,47	67,60	66,85	66,85	68,22	69,81	70,00	62,92	58,07	59,10	59,10	71,46	70,90	72,86	44,29	43,86
EsMAM1	17	104	104	104	105	99	101	103	109	108	108	100	110	134	107	106	105		77,19	77,00	70,96	76,41	75,94	75,73	75,93	75,34	75,93	75,20	75,00	71,29	72,85	70,12	73,53	66,91	74,71	74,90	74,51	73,73	73,53	75,69	72,55	73,19	74,12	69,88	69,09	68,50	68,76	75,64	75,84	68,88	63,29	65,19	64,99	71,62	71,04	73,83	44,53	44,86
BooMAM1.1	18	103	103	102	106	108	107	112	110	107	124	101	111	134	106	102	110	117		99,80	90,61	92,28	87,52	76,27	76,47	75,88	76,67	75,69	74,71	71,76	73,53	70,39	72,96	67,58	76,08	76,27	76,67	76,08	76,27	77,06	73,92	74,95	74,95	70,45	69,86	69,08	68,75	70,02	70,21	63,01	57,06	58,43	58,43	72,21	71,62	72,90	45,00	45,02
BnMAM1.1	19	104	104	103	107	109	108	113	111	108	125	102	112	135	107	103	111	118	1		90,42	92,08	87,33	76,08	76,27	75,69	76,47	75,49	74,51	71,57	73,33	70,39	72,76	67,40	76,08	76,27	76,47	75,88	76,08	76,86	73,73	74,76	74,76	70,45	69,86	69,08	68,75	70,02	70,21	63,01	57,06	58,43	58,43	72,02	71,43	72,71	45,00	45,02
BrMAM1.1	20	145	145	144	147	150	149	155	153	148	168	144	154	138	148	146	121	160	51	52		86,37	80,85	70,07	70,26	69,71	70,44	69,53	68,80	66,42	67,88	64,96	66,91	66,36	69,89	70,07	70,44	69,71	69,89	70,44	67,70	68,67	68,67	65,03	64,48	63,75	63,45	64,98	65,16	58,11	52,89	53,83	54,01	66,30	65,76	67,70	42,48	42,21
BjIPMS1	21	107	107	106	110	114	113	119	117	114	132	108	119	134	106	102	111	121	39	40	74		86,34	76,27	76,47	75,88	76,67	75,69	75,49	71,96	73,53	70,20	72,76	67,58	75,49	75,69	76,27	75,29	75,49	76,27	74,12	75,34	75,15	70,84	70,25	69,28	68,75	69,83	70,02	62,43	56,87	57,84	57,65	71,62	71,23	73,49	45,16	44,70
EvsMAM	22	124	124	124	128	131	130	132	135	135	120	127	137	158	130	127	137	121	63	64	104	69		73,37	73,57	72,98	73,77	72,19	71,99	70,22	72,19	68,24	72,89	66,67	74,07	74,26	74,07	73,48	73,48	74,26	71,79	73,03	73,23	68,64	67,85	68,05	67,32	69,88	70,08	63,84	58,22	57,34	57,14	67,72	67,52	69,22	43,44	43,68
AllMAMc.1	23	114	114	112	109	112	112	115	119	115	137	108	121	148	120	118	125	124	121	122	164	121	135		99,40	98,61	99,20	93,85	88,47	83,73	85,71	82,34	83,04	75,14	80,00	80,20	80,00	79,80	79,80	81,58	78,53	79,37	77,98	70,24	69,84	69,44	70,89	69,57	69,77	64,55	59,49	59,33	59,13	72,92	72,33	74,41	44,06	42,37
AllMAM3.2	24	113	113	111	108	111	111	114	118	114	136	107	120	147	119	117	124	123	120	121	163	120	134	3		99,20	99,40	94,05	89,07	84,13	86,31	82,74	83,43	75,51	80,20	80,40	80,20	80,00	80,00	81,78	79,13	79,96	78,17	70,83	70,04	70,04	71,49	69,77	69,96	64,95	59,49	59,33	59,13	73,12	72,53	74,61	44,38	42,68
AllMAMc.2	25	116	116	114	111	114	114	117	121	117	139	110	123	150	122	120	127	126	123	124	166	123	137	7	4		98,81	93,45	88,47	83,73	85,71	83,23	82,64	74,77	79,60	79,80	79,60	79,41	79,41	81,19	78,53	79,37	77,58	70,24	69,44	69,44	70,89	69,19	69,38	65,34	59,84	59,68	59,48	72,53	71,94	74,02	44,06	42,37
AlpMAMc	26	113	113	111	108	111	111	114	118	114	136	107	120	145	117	116	123	123	119	120	162	119	133	4	3	6		94,25	88,87	84,13	86,31	82,54	83,23	75,32	80,20	80,40	80,20	80,00	80,00	81,78	79,13	79,96	78,57	70,63	69,84	69,84	71,09	69,96	70,16	64,75	59,49	59,13	58,93	73,52	72,92	75,00	44,53	42,83
AcMAMc	27	107	107	107	102	100	100	101	118	114	138	108	120	149	121	119	126	127	124	125	167	124	141	31	30	33	29		86,31	81,94	83,93	80,36	80,31	72,78	78,26	78,46	78,06	77,87	77,67	79,05	76,39	77,03	76,04	69,11	68,32	68,12	70,36	69,05	69,25	64,03	58,98	59,21	59,01	72,73	72,13	73,62	43,28	42,21
BdMAMc	28	113	113	111	108	113	113	115	113	109	132	106	112	149	121	118	129	127	129	130	171	125	142	58	55	58	56	69		84,49	86,68	82,31	84,66	76,78	77,14	77,34	77,53	77,34	77,34	80,52	78,31	79,16	77,76	75,73	74,90	74,27	75,78	68,48	68,68	67,56	61,76	62,45	62,24	74,50	73,90	73,12	43,59	42,68
CsMAM3.1	29	125	125	124	127	129	130	130	136	136	151	132	141	165	137	134	147	147	144	145	184	143	151	82	80	82	80	91	78		94,43	86,48	82,64	76,07	75,49	75,69	75,10	74,51	74,51	77,08	77,58	78,61	75,45	69,25	68,65	68,25	67,92	65,38	65,57	60,79	55,58	55,56	55,36	69,96	69,37	70,28	43,75	43,15
CsMAM3.7	30	119	119	118	120	120	121	123	126	125	143	121	131	159	131	128	141	139	135	136	176	135	141	72	69	72	69	81	67	28		89,46	84,42	77,37	76,88	77,08	76,48	76,48	76,48	78,66	78,97	80,00	76,83	70,44	69,64	69,25	69,90	67,31	67,50	62,38	56,95	56,75	56,55	71,54	70,75	71,65	43,91	43,30
CsMAM3.3	31	133	133	132	133	134	135	137	144	142	159	137	142	176	148	141	157	153	151	151	192	152	161	89	87	84	88	99	89	68	53		79,09	72,36	73,72	73,91	73,52	73,72	73,72	75,30	73,61	74,26	72,28	66,27	65,48	65,48	66,53	66,02	66,21	65,62	59,88	60,33	60,13	67,19	66,40	67,72	42,16	41,41
CrMAM1	32	123	123	122	125	126	127	126	133	130	143	127	134	162	136	133	145	135	139	140	182	140	138	86	84	88	85	100	77	88	79	106		84,83	74,95	75,15	75,54	75,15	75,15	78,11	78,13	78,77	79,92	69,98	69,18	68,39	69,84	68,29	68,48	63,55	59,06	57,68	57,49	69,35	68,76	70,33	43,15	41,77
CrMAM3.2	33	164	164	162	164	166	166	166	168	166	181	163	168	169	173	169	164	180	177	178	185	177	181	134	132	136	133	147	124	129	122	149	81		69,94	70,13	70,87	70,13	70,50	72,73	74,21	75,00	74,95	66,36	65,61	65,23	67,16	61,79	61,97	57,65	53,60	52,90	52,71	64,33	63,59	65,06	40,95	39,64
AllMAM3.1	34	114	114	113	123	119	119	123	126	128	142	123	127	151	123	121	132	129	122	122	165	125	132	101	100	103	100	110	115	124	117	133	127	162		99,60	96,22	92,05	91,65	88,47	83,50	84,52	82,34	73,61	72,82	72,22	72,08	70,23	70,43	64,36	58,32	57,94	57,74	72,78	72,19	73,87	43,66	44,15
AlpMAMb	35	114	114	113	122	118	118	122	125	127	141	122	126	150	122	120	131	128	121	121	164	124	131	100	99	102	99	109	114	123	116	132	126	161	2		96,61	92,05	91,65	88,47	83,50	84,52	82,34	73,81	73,02	72,42	72,28	70,43	70,62	64,55	58,51	58,13	57,94	72,98	72,39	74,07	43,82	44,31
AcMAMb	36	117	117	116	126	122	122	126	124	124	139	119	124	146	117	115	132	130	119	120	162	121	132	101	100	103	100	111	113	126	119	134	124	157	19	17		93,64	93,24	88,47	83,10	83,93	82,94	73,41	72,62	71,83	71,88	70,62	70,82	65,15	58,51	58,53	58,33	73,18	72,39	74,07	43,97	44,46
AtMAM3	37	116	116	115	129	124	124	128	123	122	137	120	126	151	123	122	135	134	122	123	166	126	135	102	101	104	101	112	114	129	119	133	126	161	40	40	32		99,20	88,67	83,50	84,13	82,34	72,82	72,02	71,43	70,89	70,04	70,23	64,75	58,71	58,33	57,94	73,18	72,58	74,66	43,44	43,61
AtsMAM3	38	117	117	116	130	125	125	129	122	121	136	121	126	152	124	123	136	135	121	122	165	125	135	102	101	104	101	113	114	129	119	133	126	159	42	42	34	4		88,47	83,30	83,93	82,34	72,82	72,02	71,43	70,89	70,04	70,23	64,75	58,51	58,13	57,74	73,18	72,58	74,66	43,44	43,61
BdMAMb	39	105	105	104	114	111	110	114	119	115	131	109	117	146	118	119	128	124	117	118	162	121	131	93	92	95	92	106	98	116	108	125	111	147	58	58	58	57	58		87,08	88,29	84,72	73,41	72,82	72,62	71,68	70,82	71,01	65,35	59,69	58,73	58,53	72,98	72,39	74,26	43,12	43,15
CsMAM3.2	40	124	124	123	130	135	135	137	139	138	154	133	143	160	132	130	149	140	133	134	177	132	143	108	105	108	105	119	108	113	106	133	110	138	83	83	85	83	84	65		96,79	89,18	73,75	73,15	72,95	71,20	66,73	66,93	63,00	58,30	56,31	56,11	71,43	70,83	72,92	43,28	43,30
CsMAM3.8	41	116	116	115	123	127	127	129	133	132	149	128	138	156	128	124	146	137	128	129	172	126	137	104	101	104	101	116	104	108	101	130	107	134	78	78	81	80	81	59	16		90,60	74,00	73,40	73,00	71,06	67,96	68,16	63,47	58,97	57,40	57,20	72,48	71,88	74,16	43,84	43,70
CrMAM3.1	42	123	123	123	129	131	131	132	138	135	153	132	140	157	129	127	147	132	128	129	172	127	136	111	110	113	108	121	111	124	117	140	101	134	89	89	86	89	89	77	54	47		74,00	73,20	72,80	69,86	67,70	67,90	63,07	58,58	57,20	57,00	70,10	69,70	72,33	43,75	43,61
CsMAM3.4	43	142	142	141	147	153	153	155	156	156	168	152	157	190	162	160	173	153	151	151	192	149	159	150	147	150	148	156	117	155	149	170	151	180	133	132	134	137	137	134	131	130	130		99,15	95,73	81,65	64,01	64,20	63,81	57,97	58,28	58,07	69,08	68,27	67,59	41,85	41,81
CsMAM3.5	44	144	144	143	149	157	157	159	160	160	172	156	161	194	166	164	177	157	154	154	195	152	163	152	151	154	152	160	121	158	153	174	155	184	137	136	138	141	141	137	134	133	134	4		94,87	80,80	63,42	63,62	63,18	57,56	58,49	58,28	68,67	67,87	67,19	41,54	41,50
CsMAM3.6	45	149	149	148	150	158	158	160	161	161	171	158	162	196	168	167	177	160	158	158	199	157	162	154	151	154	152	161	124	160	155	174	159	186	140	139	142	144	144	138	135	135	136	20	24		82,28	63,23	63,42	62,97	57,14	56,39	56,18	67,87	67,47	66,40	41,22	41,34
CrMAM3.3	46	144	144	142	145	150	149	150	149	150	162	146	150	189	160	158	170	159	160	160	201	160	166	147	144	147	146	150	117	162	152	169	152	176	141	140	142	147	147	143	144	145	151	87	91	84		65,56	65,76	65,69	59,63	58,79	58,58	69,48	69,08	68,97	42,41	41,81
BnMAM1.2	47	141	141	141	147	147	147	148	149	146	148	142	152	173	144	146	163	123	155	155	194	156	153	157	156	159	155	160	162	179	169	174	163	209	153	152	151	154	154	150	171	165	166	185	188	189	177		99,80	84,10	70,92	64,66	64,46	64,85	64,27	66,80	43,82	43,53
BrMAM1.2	48	140	140	140	146	146	146	147	148	145	148	141	151	172	143	145	162	122	154	154	193	155	152	156	155	158	154	159	161	178	168	173	162	208	152	151	150	153	153	149	170	164	165	184	187	188	176	1		83,94	70,78	64,53	64,33	65,05	64,47	66,99	43,97	43,68
BrpMAM.1	49	173	173	173	176	178	178	179	178	175	172	171	181	201	170	173	198	155	189	189	230	192	179	179	177	174	178	182	157	198	190	165	183	227	180	179	176	178	178	175	185	183	185	173	176	177	164	79	80		80,79	74,65	74,41	61,17	60,76	61,58	39,03	38,34
RsMAM1.2	50	201	201	201	199	201	201	200	205	201	201	195	206	230	200	201	226	185	222	222	261	223	211	207	207	204	207	210	187	227	220	195	208	251	213	212	212	211	212	206	211	208	210	203	205	207	195	146	147	83		75,24	75,00	56,46	56,06	57,34	36,66	35,93
BrMAM1.4	51	198	198	198	195	197	196	199	198	195	197	192	201	227	196	192	218	173	212	212	253	215	212	205	205	202	206	206	181	224	218	190	212	252	212	211	209	210	211	208	218	213	214	199	198	208	197	176	177	108	103		99,50	57,23	56,83	57,51	35,16	35,11
BrpMAM.2	52	199	199	199	196	198	197	200	198	195	197	193	202	228	197	193	218	174	212	212	252	216	213	206	206	203	207	207	182	225	219	191	213	253	213	212	210	212	213	209	219	214	215	200	199	209	198	177	178	109	104	2		57,03	56,63	57,31	35,01	34,95
BooMAM1.2	53	121	121	120	126	126	128	132	133	132	154	127	142	172	144	143	153	145	142	143	185	145	164	137	136	139	134	138	127	152	144	166	156	193	138	137	136	136	136	137	144	139	151	154	156	160	152	181	180	193	219	213	214		98,19	88,91	44,84	44,24
BrMAM1.3	54	123	123	122	128	128	130	134	136	135	157	131	145	174	146	145	156	148	145	146	188	147	165	140	139	142	137	141	130	155	148	170	159	197	141	140	140	139	139	140	147	142	153	158	160	162	154	184	183	195	221	215	216	9		88,12	45,47	43,93
RsMAM1.1	55	122	122	121	127	125	126	129	133	131	151	130	140	167	139	134	146	134	139	140	178	136	157	130	129	132	127	134	136	151	144	164	151	189	133	132	132	129	129	131	137	131	140	164	166	170	157	171	170	194	218	215	216	56	60		45,94	45,48
AtIPMS1	56	350	350	350	354	352	351	354	348	348	356	347	350	379	351	350	371	355	352	352	390	351	362	358	356	358	355	363	361	360	359	369	365	398	360	359	358	362	362	364	363	360	360	371	373	375	368	359	358	389	406	413	414	353	349	346		86,28
AtIPMS2	57	355	355	355	360	356	355	358	356	356	359	352	357	385	357	352	375	354	353	353	393	355	361	370	368	370	367	371	368	365	364	375	375	408	358	357	356	362	362	365	364	362	362	373	375	376	373	362	361	394	412	414	415	358	360	350	87	

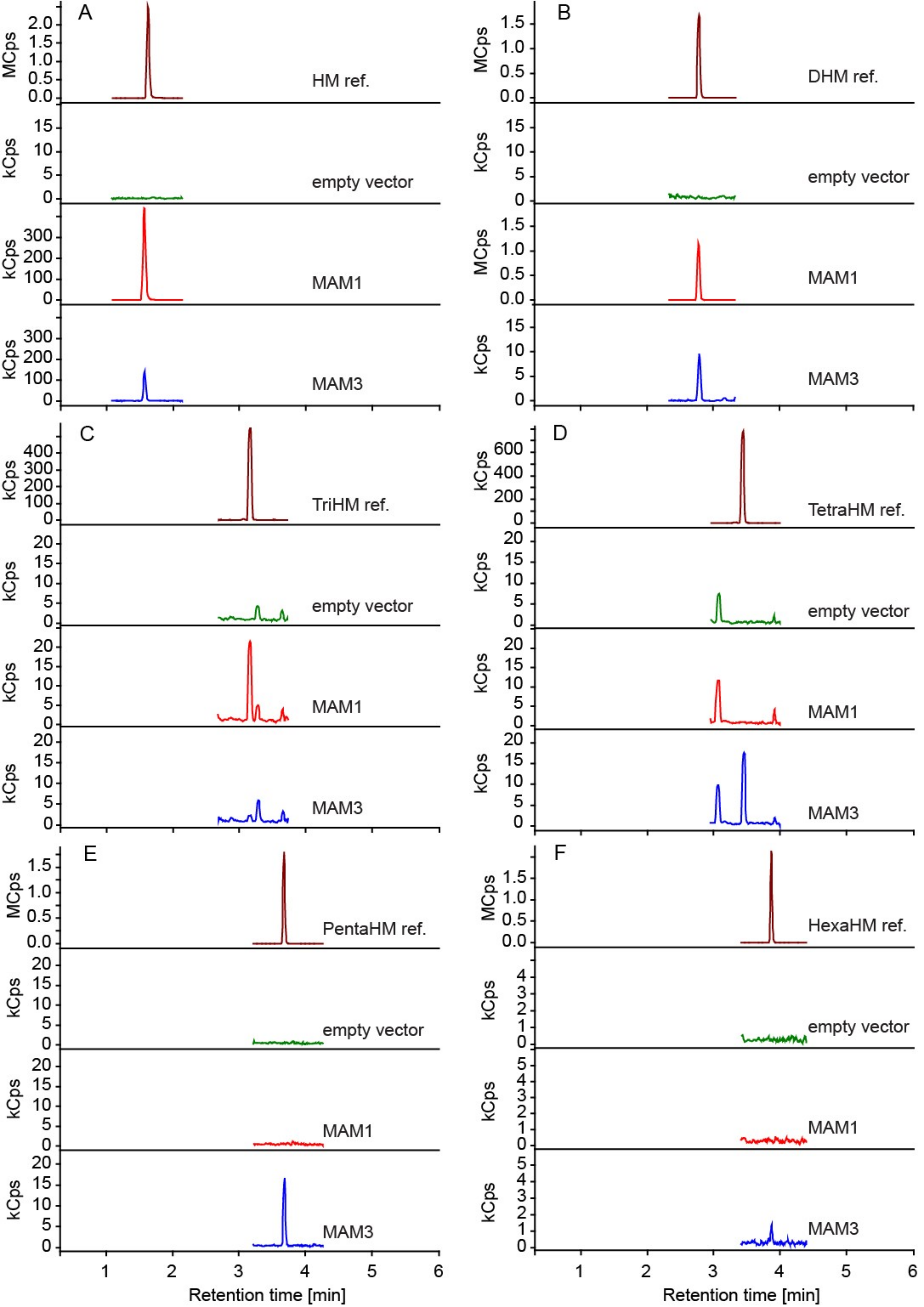
A
HM ref.
empty vector
MAM1
MAM3
B
DHM ref.
empty vector
MAM1
MAM3
C
TriHM ref.
empty vector
MAM1
MAM3
D
TetraHM ref.
empty vector
MAM1
MAM3
E
PentaHM ref.
empty vector
MAM1
MAM3
F
HexaHM ref.
empty vector
MAM1
MAM3
MCps
kCps
Retention time [min]

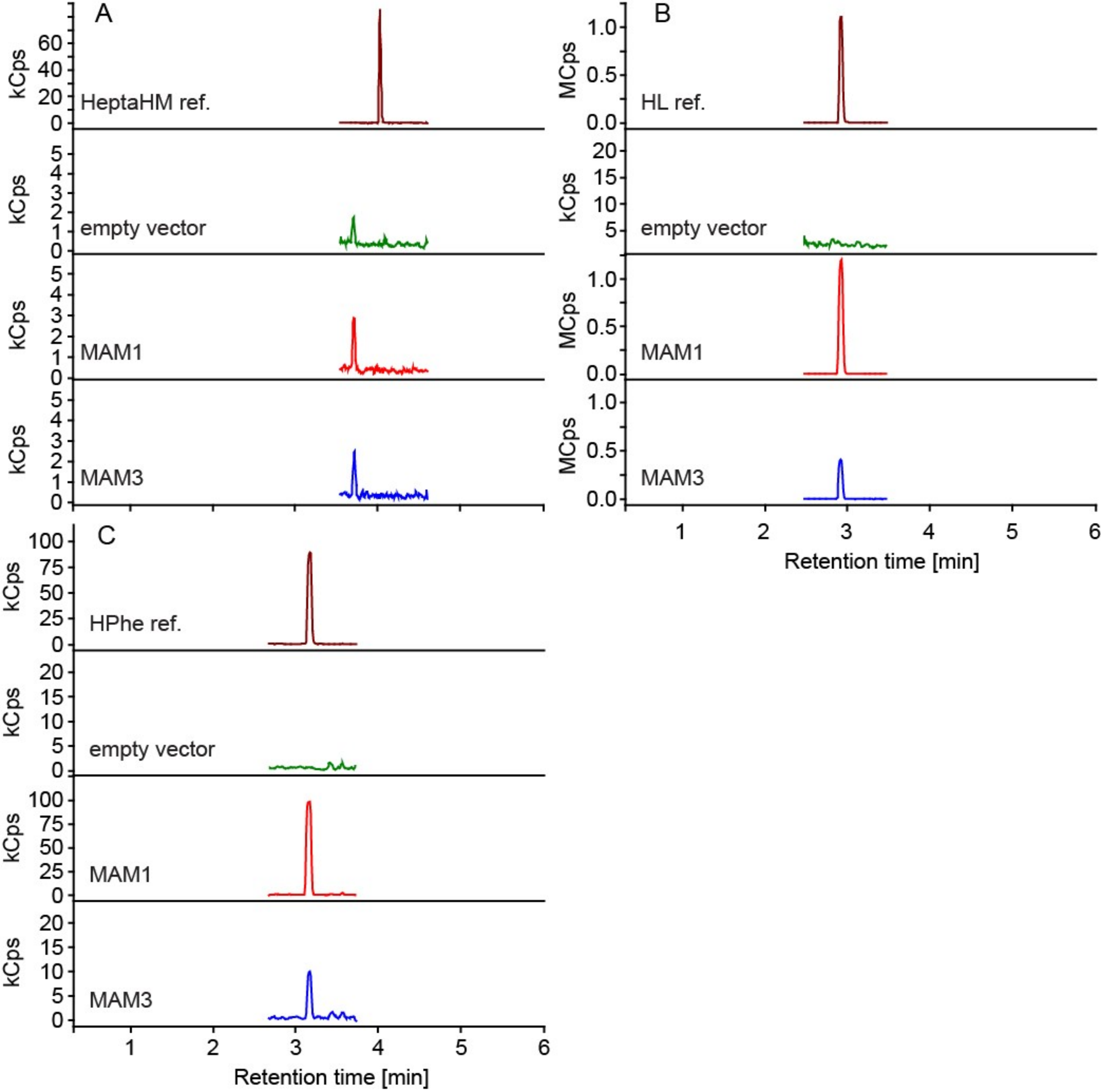

A
kCps
HeptaHM ref.
empty vector
MAM1
MAM3
B
MCps
HL ref.
empty vector
MAM1
MAM3
Retention time [min]
C
kCps
HPhe ref.
empty vector
MAM1
MAM3
Retention time [min]

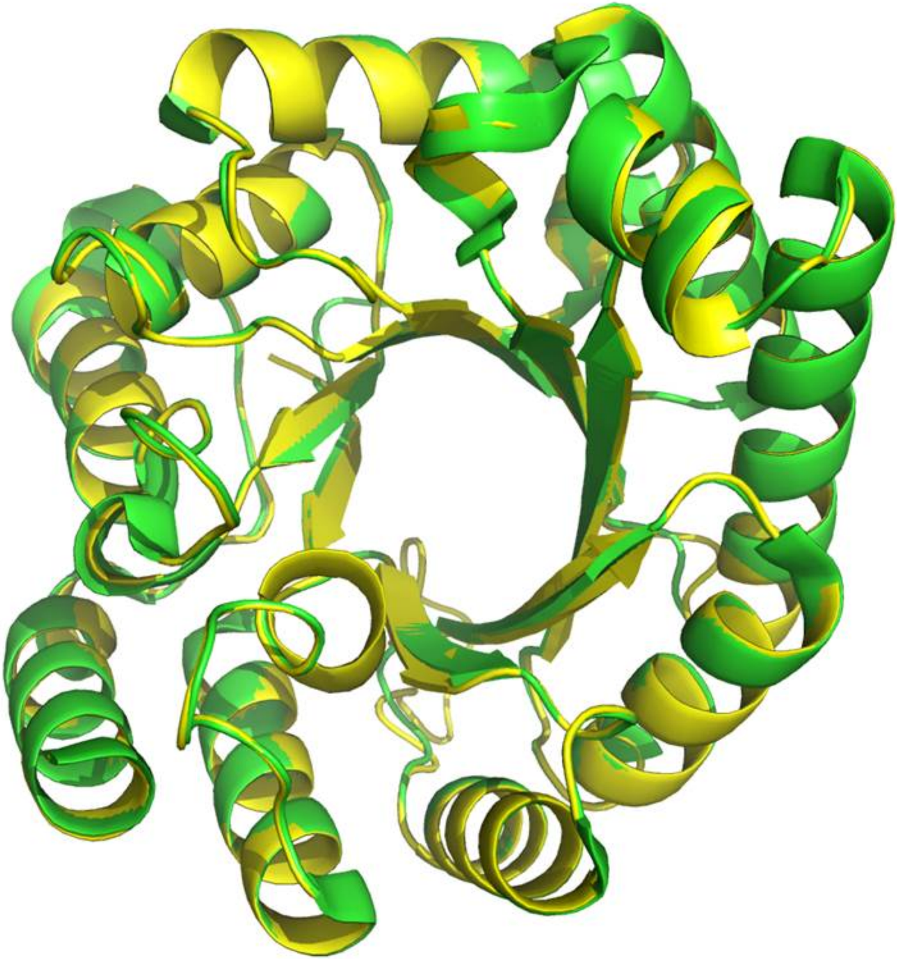

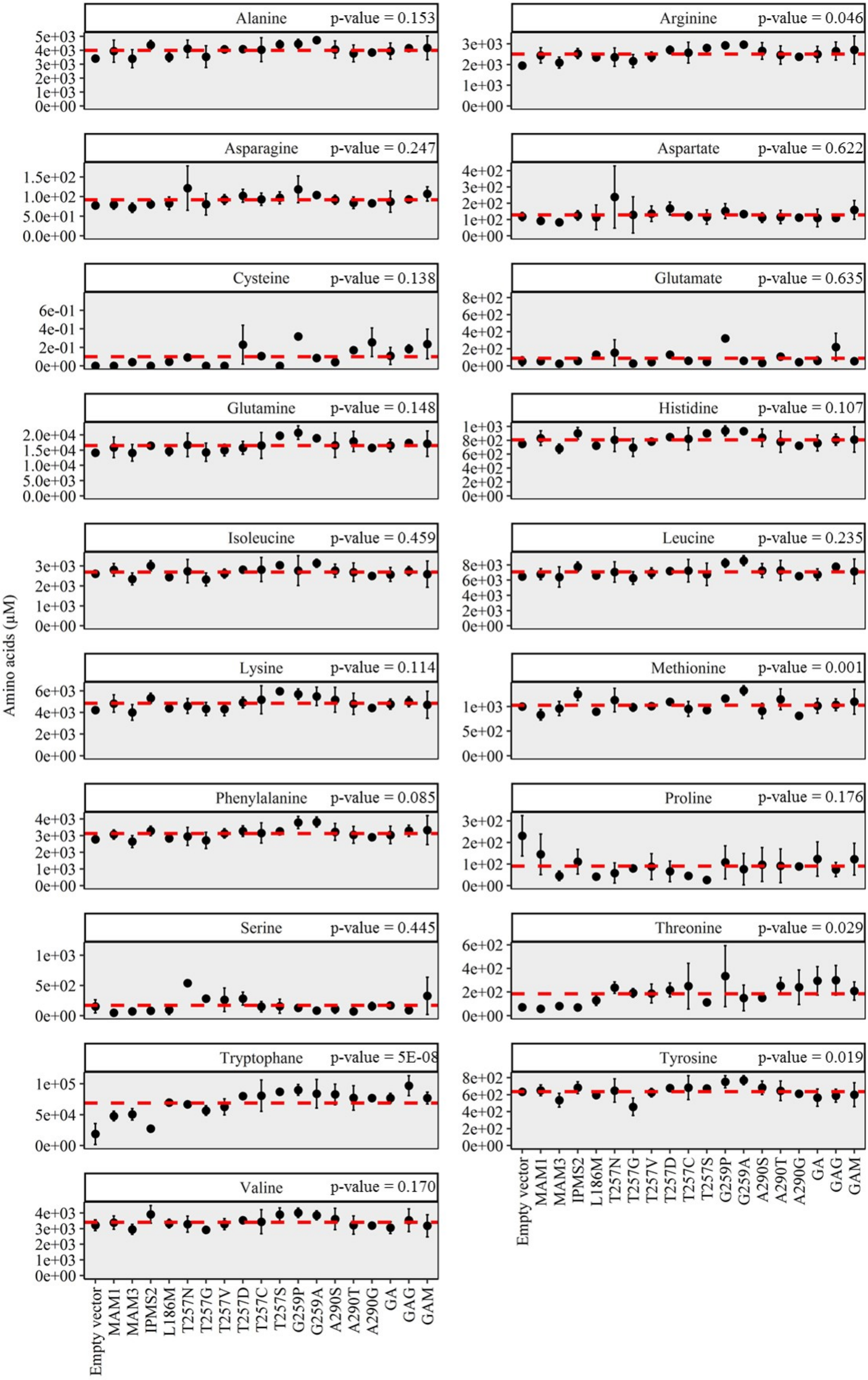

Alanine p-value = 0.153
Arginine p-value = 0.046
Asparagine p-value = 0.247
Aspartate p-value = 0.622
Cysteine p-value = 0.138
Glutamate p-value = 0.635
Glutamine p-value = 0.148
Histidine p-value = 0.107
Isoleucine p-value = 0.459
Leucine p-value = 0.235
Lysine p-value = 0.114
Methionine p-value = 0.001
Phenylalanine p-value = 0.085
Proline p-value = 0.176
Serine p-value = 0.445
Threonine p-value = 0.029
Tryptophane p-value = 5E-08
Tyrosine p-value = 0.019
Valine p-value = 0.170
Amino acids (µM)
Empty vector
MAM1
MAM3
IPMS2
L186M
T257N
T257G
T257V
T257D
T257C
T257S
G259P
G259A
A290S
A290T
A290G
GA
GAG
GAM

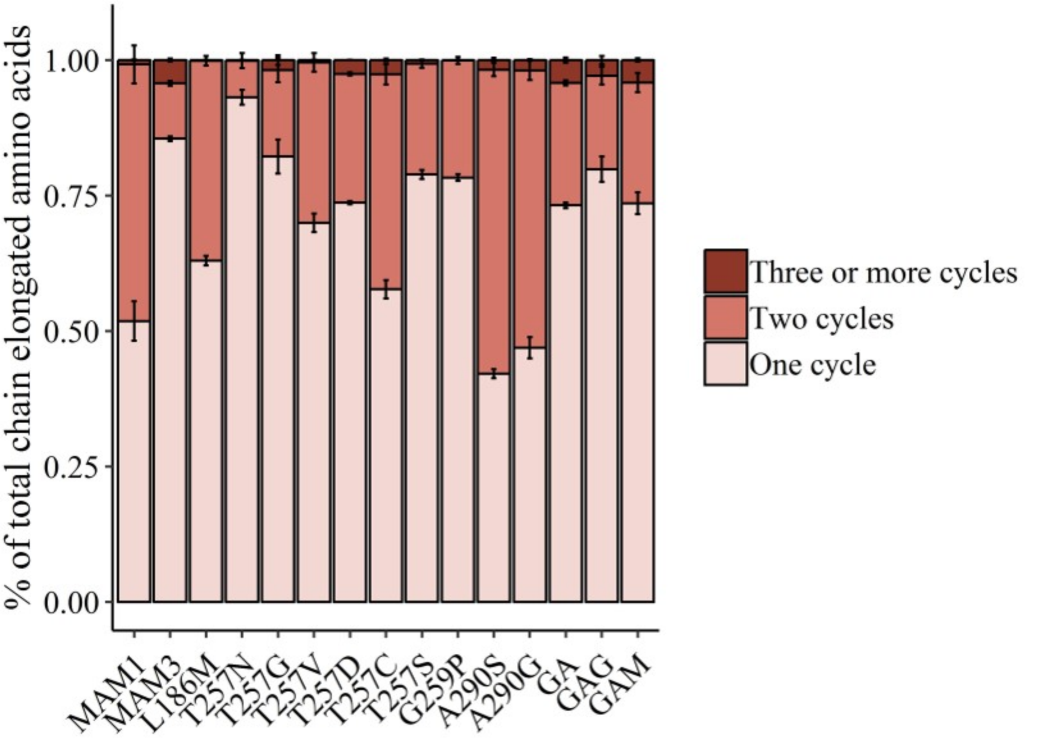

% of total chain elongated amino acids
1.00
0.75
0.50
0.25
0.00
MAM1
MAM3
L186M
T257N
T257G
T257V
T257D
T257C
T257S
G259P
A290S
A290G
GA
GAG
GAM
Three or more cycles
Two cycles
One cycle

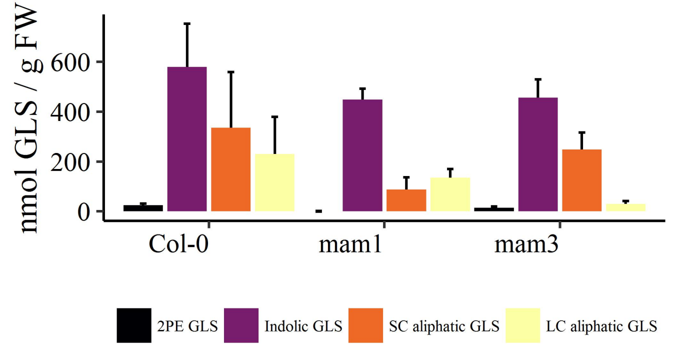
nmol GLS / g FW
600
400
200
0
Col-0
mam1
mam3
2PE GLS
Indolic GLS
SC aliphatic GLS
LC aliphatic GLS

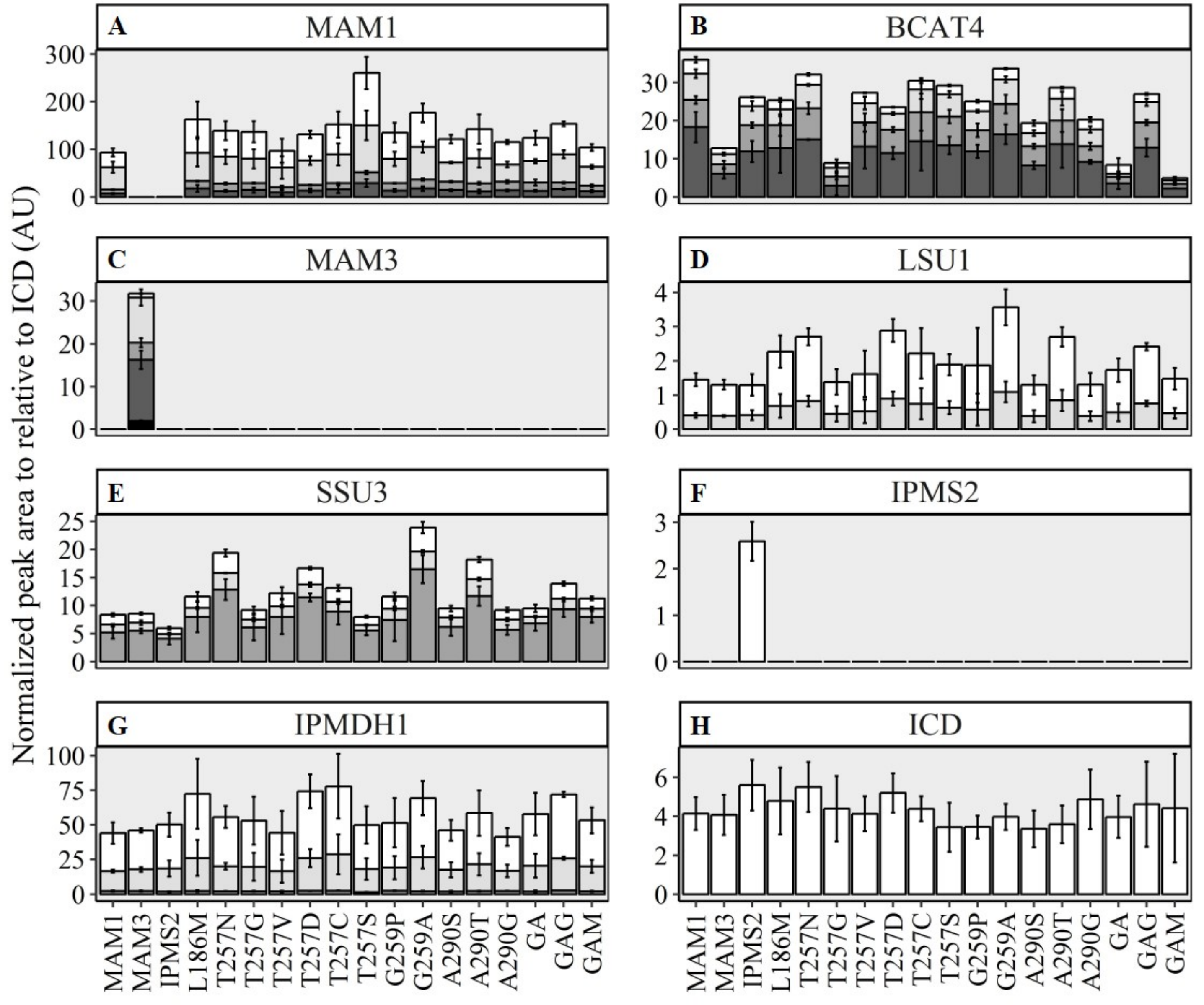

Normalized peak area to relative to ICD (AU)
A
MAM1
B
BCAT4
C
MAM3
D
LSU1
E
SSU3
F
IPMS2
G
IPMDH1
H
ICD
MAM1
MAM3
IPMS2
L186M
T257N
T257G
T257V
T257D
T257C
T257S
G259P
G259A
A290S
A290T
A290G
GA
GAG
GAM

Supplementary Figure legends

Supplementary Figure S1. Alignment of 57 MAM and 2 IPMS full protein sequences. According to *A. thaliana* MAM1 numbering, only Leu186, Thr257, Ile258, Gly259 and Ala290 residues are shown.

Supplementary Figure S2. Matrix from the alignment of MAM and IPMS enzyme sequences. Upper part indicates sequence similarity in percentage and the lower part shows differences between the protein sequences. Two MAM sequences have been removed from the matrix, as the comparison indicated low sequence similarity due to poor quality of the sequence data.

Supplementary Figure S3. LC-MS traces of homomethionine (HM), dihomomethionine (DHM), trihomomethionine (TriHM), tetrahomomethionine (TetraHM), pentahomomethionine (PentaHM) and hexahomomethionine (HexaHM). A representative sample of MAM1 and MAM3 containing strains as well as an empty vector control are shown. For each compound, the pure sample is shown in the top panel.

Supplementary Figure S4. LC-MS traces of heptahomomethionine (HeptaHM), homoleucine (HL) and homophenylalanine (HPhe). A representative sample of MAM1 and MAM3 containing strains as well as an empty vector control are shown. For each compound, the pure sample is shown in the top panel.

Supplementary Figure S5. Homology model of MAM1 from *A. thaliana*. (yellow) superimposed on the crystal structure of *N. meningitis* IPMS (green). Only the $(\beta/\alpha)_8$ catalytic barrel is shown. The RMSD value is 0.26 Å.

Supplementary Figure S6. Protein amino acids monitored in this study. Each graph shows levels of one amino acid in the media of *E. coli* strains expressing MAM variants and an empty vector control. The mean of all cultures is indicated by the red line. ANOVA was used to investigate changes in the individual amino acids between the cultures. p-values are shown in upper right corner of each graph. Data represents average and standard deviation of three biological replicates each grown in three technical replicates.

Supplementary Figure S7. Produced chain elongated amino acids were grouped according to how many methylene groups were added to the methionine, phenylalanine or leucine structures (one, two or three and above). Bars represents the percentage of each group of products relative to the total production within each culture. Data represents average and standard deviation of three biological replicates each grown in three technical replicates.

Supplementary Figure S8. Glucosinolate analysis in *A. thaliana* ecotype Col-0 and knockout mutants mam1 and mam3. Glucosinolates (GLS) were grouped into aromatic (2PE) GLSs, indole GLSs and short chain (SC – one to three elongation rounds) and long chain (LC – four elongation rounds and above) aliphatic GLSs. Data represents average and standard deviation of 3-7 biological replicates.

Supplementary Figure S9. Protein levels in *E. coli* strains expressing the chain elongation pathway genes, A) *BCAT4,* B) *MAM1,* C) *LSU1,* D) *MAM3,* E) *SSU3,* F) *IPMS2* and G) *IPMDH1* and H) *E. coli* housekeeping gene *ICD.* A-G are normalized the ICD, while the ICD is not normalized. Different colours indicate individual peptides within each protein. Data represents average and standard deviation of three biological replicates.

Supplementary Tables

Supplementary Table S1. List of primers used in this study. Lower case letters denotes the USER tail used for cloning. Uracils are marked in blue. Mutated nucleotides are marked in red.

**Name**	**Sequence**	***Description***
MAM1_fwd	ggcttaauATGCATCACCACCACCATCACAACTATGTGCGTGTATTC	*MAM1* forward primer
BCAT4_rev	ggtttaauTCAGCCCTGGCGGTCAAT	*BCAT4* reverse primer
MAM3_fwd	ggcttaauATGCATCACCACCACCATCACGCTGAGTCCAAAAAGGTGGC	*MAM3* forward primer
MAM3_rev	acaaaattauTTCTAGAGGGGTTATACAACAGCGGAAATC	*MAM3* reverse primer
IPMS2_fwd	ggcttaauATGCATCACCACCACCATCACATGGAGTCTTCGATTCTCA	*IPMS2* forward primer
IPMS2_rev	acaaaattauTTCTAGAGGGGTCAGGCAGGGACTTCGTTG	*IPMS2* reverse primer
L186_fwd	acatctactaguGACATTCACATGAAATATAAG	Fusion primer for *L186*-mutant
L186M_rev	actagtagatguGAATACCATTATCCTTGGCCTC	Fusion primer for *L186M*-mutant
T257/G259_fwd	acggtagggauCAACATGCCACATGAATACGG	Fusion primer for *T257*-mutant
T257N_rev	atccctaccguATCACCGATGTTCACCACCGTAA	Fusion primer for *T257N*-mutant
T257G_rev	atccctaccguATCACCGATGCCCACCACCGTAA	Fusion primer for *T257G*-mutant
T257V_rev	atccctaccguATCACCGATGACCACCACCGTAA	Fusion primer for *T257V*-mutant
T257D_rev	atccctaccguATCACCGATGTCCACCACCGTAA	Fusion primer for *T257D*-mutant
T257C_rev	atccctaccguATCACCGATGCACACCACCGTAA	Fusion primer for *T257C*-mutant
T257S_rev	atccctaccguATCACCGATGCTCACCACCGTAA	Fusion primer for *T257S*-mutant
G259P_rev	atccctaccguATCTGGGATGGTCACCACCGTAA	Fusion primer for *G259P*-mutant
G259A_rev	atccctaccguATCGGCGATGGTCACCACCGTAA	Fusion primer for *G259A*-mutant
A290S_fwd	acgatgttgtcguCAGCGTTCATTGTCACAACGAC	Fusion primer for *A290S*-mutant
A290T_fwd	acgatgttgtcguCACCGTTCATTGTCACAACGAC	Fusion primer for *A290T*-mutant
A290G_fwd	acgatgttgtcguCGGCGTTCATTGTCACAACGAC	Fusion primer for *A290G*-mutant
A290_rev	acgacaacatcguCAATTCCAGGGGTGTTTGCTTTG	Fusion primer for *A290*-mutant
GA_rev	atccctaccguATCGGCGATGCCCACCACCGTAA	Fusion primer for *T257G/G259A*-mutant

Supplementary Table S2. Transitions and response factors used to quantify levels of chain elongated amino acids. Response factors of methionine- and leucine-derived products were reported in (20).

Amino acid	Q1	Q3	CE	Internal standard	Q1	Q3	Response factor
HPhe	180.1	91.1 Qt	-22	13C,15N-Phe	176.2	129.2	1.61
	180.1	134.1	-8				
	180.1	117.1	-13				
HM	164.2	70.3 Qt	-16	13C,15N-Val	124.1	77.2	0.35
DHM	178.2	84.2 Qt	-14	13C,15N-Phe	176.2	129.2	1.05
	178.2	132.1	-10				
TriHM	192.2	98.2 Qt	-14	13C,15N-Phe	176.2	129.2	1.25
	192.2	146.1	-9				
TetraHM	206.2	112.2 Qt	-13	13C,15N-Phe	176.2	129.2	0.91
	206.2	160.1	-10				
PentaHM	220.3	126.2 Qt	-16	13C,15N-Phe	176.2	129.2	0.41
	220.3	174.1	-10				
HexaHM	234.4	140.2 Qt	-16	13C,15N-Phe	176.2	129.2	0.36
	234.4	188.1	-11				
HeptaHM	248.2	188.1 Qt	-10	13C,15N-Phe	176.2	129.2	n.d.
	148.2	154.1	-16				
HL	146.2	100.1	10	13C,15N-Phe	176.2	129.2	0.88
DHL	160.2	114.1	-10	13C,15N-Phe	176.2	129.2	1.00*
TriHL	174.2	128.1	-10	13C,15N-Phe	176.2	129.2	1.00*

*Response factors for DHL and TriHL were assumed to be 1.00. CE = collision energy. Q = quadrupole. Qt = quantifier transition

Supplemental Table S3: Transitions used for Selected Reaction Monitoring (SRM) for targeted proteomics on peptides of methionine chain elongation pathway proteins. Retention times and parent ion to fragment ion transitions are given. All peptides were selected in Q1 as the double-charged ions $[M+H]^{2+}$ and fragment ions in Q3 as single-charged ions $[M+H]^{+}$. Collision energies (CE) for natural non-labelled (light) and isotopically labelled (heavy) peptides were identical.

				Light peptide		Heavy peptide		
Protein name	AGI code	Peptide	RT	Q1 $[M+H]^{2+}$	Q3 $[M+H]^{+}$	Q1 $[M+H]^{2+}$	Q3 $[M+H]^{+}$	CE [eV]
BCAT4	At3g19710	TGEETLAAK	1.60	460.25	761.42	464.25	769.42	-13
			1.60	460.25	818.44	464.25	826.44	-15
			1.60	460.25	632.38	464.25	640.38	-14
		LYETLSDIQTGR*	8.30	698.36	776.4	703.36	786.4	-19
			8.30	698.36	990.53	703.36	1000.53	-26
			8.30	698.36	1119.57	703.36	1129.57	-23
		SITNYCPVWIPLAEAK	18.70	931.49	927.54	935.49	935.54	-3
			18.70	931.49	1123.67	935.49	1131.67	-29
			18.70	931.49	628.38	935.49	636.38	-41
		GNVVSTPTIAGTILPGVTR	16.50	927.03	529.32	932.03	539.32	-29
			16.50	927.03	642.4	932.03	652.4	-28
MAM1	At5g23010	SLGFNDIQFGCEDGGR*	13.60	886.39	897.36	891.39	907.36	-31
			13.60	886.39	1025.42	891.39	1035.42	-33
			13.60	886.39	404.2	891.39	414.2	-41
		DGEQSPGGSLTPPQK	2.50	749.37	981.55	753.37	989.55	-23
			2.50	749.37	1068.58	753.37	1076.58	-25
			2.50	749.37	469.29	753.37	477.29	-30
		STYEILSPEDIGIVK	17.00	832.45	957.54	836.45	965.54	-22
			17.00	832.45	870.51	836.45	878.51	-25
			17.00	832.45	1070.62	836.45	1078.62	-24
		SGNASLEEVVMALK	17.50	724.38	918.51	728.38	1126.63	-24
			17.50	724.38	1118.63	728.38	1039.59	-24
			17.50	724.38	1031.59	728.38	926.51	-23
MAM3	At5g23020	GESLMDGVYTK	6.70	600.29	813.4	604.29	821.4	-19
			6.70	600.29	1013.51	604.29	1021.51	-16
			6.70	600.29	682.35	604.29	690.35	-19
		ALVVNGAEISSEK	5.70	658.86	934.46	662.86	942.46	-20
			5.70	658.86	1033.53	662.86	1041.53	-20
			5.70	658.86	820.42	662.86	828.42	-21
		SGNAPLEEVVMALK	18.70	729.39	1128.65	733.39	1136.65	-21
			18.70	729.39	918.51	733.39	926.51	-25
			18.70	729.39	588.4	733.39	596.4	-23
		STYEILSPEDVGIVK*	15.50	825.44	943.52	829.44	951.52	-23
			15.50	825.44	856.49	829.44	864.49	-23
			15.50	825.44	1056.61	829.44	1064.61	-25
		DGEQSPGAALTPPQK	3.40	748.38	469.29	752.38	477.29	-32
			3.40	748.38	372.24	752.38	380.24	-41

			3.40	748.38	979.57	752.38	987.57	-24
IPMS2	At1g74040	GTYEIMSPEEIGLER*	15.40	889.94	1102.53	894.94	1112.53	-28
			15.40	889.94	942.5	894.94	952.5	-32
IPMI-LSU1	At4g13430	FILDGEMPSYLQAK*	16.90	806.42	806.45	810.42	814.45	-26
			16.90	806.42	937.5	810.42	945.5	-24
			16.90	806.42	1123.56	810.42	1131.56	-24
		VWMDVYALPVPGAGGK	18.50	830.44	682.4	834.44	690.4	-21
			18.50	830.44	486.28	834.44	494.28	-37
			18.50	830.44	261.17	834.44	269.17	-49
IPMI-SSU3	At3g58990	LGSFALNGLPK	12.60	558.83	712.45	562.83	720.45	-19
			12.60	558.83	1003.57	562.83	1011.57	-18
			12.60	558.83	641.41	562.83	649.41	-17
		EDGSSLLINHTTR	3.90	721.87	854.49	726.87	864.49	-29
			3.90	721.87	967.58	726.87	977.58	-29
			3.90	721.87	741.41	726.87	751.41	-28
		NCVATGEIFPLESEVR*	15.10	910.95	829.45	915.95	839.45	-30
			15.10	910.95	976.52	915.95	986.52	-30
			15.10	910.95	490.27	915.95	500.27	-37
IPMDH1	At5g14200	LSDAILLGAIGGYK*	17.00	695.91	891.54	699.91	899.54	-19
			17.00	695.91	778.46	699.91	786.46	-20
			17.00	695.91	1004.63	699.91	1012.63	-22
		AGSLEGLEFDFK	14.20	656.83	855.44	660.83	863.44	-20
			14.20	656.83	984.48	660.83	992.48	-18
			14.20	656.83	685.33	660.83	693.33	-17
		IEDAVVDALNK	6.05	593.83	944.52	597.83	952.52	-17
			6.05	593.83	659.39	597.83	667.39	-17
			6.05	593.83	758.45	597.83	766.45	-17
ICD	JW1122	GPLTTPVGGGIR*	6.37	562.83	655.40	567.83	665.40	-20
			6.37	562.83	857.49	567.83	867.49	-21
			6.37	562.83	756.44	567.83	766.44	-19

*= peptides represented in Figure 8.

Supplemental Table S4: Pairwise t-test of the methionine levels from media of *E. coli* strains expression the chain elongation pathway with 18 different MAM variants and an empty vector control. p-values shown.

	Empty vector	MAM1	MAM3	IPMS2	L186M	T257N	T257G	T257V	T257D	T257C	T257S	G259P	G259A	A290S	A290T	A290G	GA	GAG
MAM1	0.1316	-	-	-	-	-	-	-	-	-	-	-	-	-	-	-	-	-
MAM3	0.7101	0.2507	-	-	-	-	-	-	-	-	-	-	-	-	-	-	-	-
IPMS2	0.0254	0.0004	0.5510	-	-	-	-	-	-	-	-	-	-	-	-	-	-	-
L186M	0.3535	0.0103	0.5755	0.0023	-	-	-	-	-	-	-	-	-	-	-	-	-	-
T257N	0.2301	0.0088	0.1192	0.2755	0.0372	-	-	-	-	-	-	-	-	-	-	-	-	-
T257G	0.8806	0.1727	0.8245	0.0178	0.4355	0.1784	-	-	-	-	-	-	-	-	-	-	-	-
T257V	0.9379	0.1136	0.6532	0.0305	0.3152	0.2609	0.8196	-	-	-	-	-	-	-	-	-	-	-
T257D	0.3792	0.0199	0.2139	0.1591	0.0752	0.7432	0.3045	0.4223	-	-	-	-	-	-	-	-	-	-
T257C	0.6634	0.2773	0.9491	0.0087	0.6195	0.1055	0.7753	0.6080	0.1920	-	-	-	-	-	-	-	-	-
T257S	0.5230	0.3757	0.7885	0.0051	0.7698	0.0700	0.6245	0.4740	0.1332	0.8379	-	-	-	-	-	-	-	-
G259P	0.1362	0.0040	0.0655	0.4266	0.0185	0.7637	0.1024	0.1569	0.5307	0.0572	0.0366	-	-	-	-	-	-	-
G259A	0.0043	4.9e-05	0.0016	0.4823	0.0003	0.0773	0.0029	0.0053	0.0383	0.0013	0.0007	0.1385	-	-	-	-	-	-
A290S	0.4230	0.4693	0.6657	0.0033	0.8977	0.0494	0.5140	0.3799	0.0974	0.7125	0.8695	0.0251	0.0005	-	-	-	-	-
A290T	0.1735	0.0057	0.0862	0.3536	0.0254	0.8680	0.1323	0.1985	0.6218	0.0758	0.0492	0.8930	0.1075	0.0342	-	-	-	-
A290G	0.0920	0.8519	0.1836	0.0002	0.4347	0.0054	0.1229	0.0786	0.0126	0.2048	0.2851	0.0024	2.8e-05	0.3639	0.0035	-	-	-
GA	0.8826	0.0993	0.6039	0.0357	0.2834	0.2909	0.7659	0.9444	0.4632	0.5604	0.4326	0.1776	0.0064	0.3438	0.2232	0.0681	-	-
GAG	0.7299	0.0666	0.4745	0.0552	0.2058	0.3888	0.6206	0.7891	0.5911	0.4364	0.3272	0.2475	0.0106	0.2541	0.3053	0.0446	0.8432	-
GAM	0.3673	0.0189	0.2059	0.1656	0.0718	0.7602	0.2943	0.4096	0.9821	0.1847	0.1278	0.5454	0.0403	0.0931	0.6377	0.0119	0.4498	0.5758
